# Structural characterization of an α-glucosaccharide-binding protein from *Paenibacillus* sp. str. FPU-7

**DOI:** 10.1016/j.yjsbx.2026.100149

**Published:** 2026-05-28

**Authors:** Takafumi Itoh, Kanato Kataoka, Yuma Kaneko, Takao Hibi, Hisashi Kimoto

**Affiliations:** Department of Bioscience and Biotechnology, Fukui Prefectural University, 4-1-1, Matsuokakenjyojima, Eiheiji-cho, Yoshida-gun, Fukui, 910-1195, Japan

**Keywords:** ABC transporter, α-Glucosaccharide, Maltose-binding protein, *Paenibacillus*, Solute-binding protein

## Abstract

*Paenibacillus* sp. str. FPU-7 (*P*. FPU-7), a chitinolytic bacterium, efficiently degrades chitin and uses the solute-binding proteins (SBPs) NagB1 and NagB2 on the cell surface to facilitate the uptake and intracellular transport of chitooligosaccharides. SBPs are essential components of the bacterial carbohydrate transport system and play key roles in carbon source acquisition. However, they exhibit diverse substrate specificities with ligands yet to be identified. Predicting SBP ligands solely from amino acid sequences remains a significant challenge. In addition to encoding NagB1 and NagB2, the genome of *P*. FPU-7 encodes several SBPs that are potentially involved in carbohydrate import. In the present study, we identified a novel SBP, designated PsMBP, in the *P*. FPU-7 genome and confirmed its mRNA expression. Genes adjacent to *psmbp* encode transmembrane domains, suggesting that PsMBP functions as part of an ABC transporter complex. We also characterized the sugar-binding specificity of PsMBP using biochemical analysis. PsMBP exhibited binding affinities for various α-glucosaccharides, including maltose, trehalose, isomaltose, sucrose, maltotriose, and maltotetraose. In particular, it showed high binding affinity for both maltose and trehalose. Furthermore, we determined the crystal structure of PsMBP in its ligand-free form and in complexes with different saccharides at resolutions of 1.4–2.1 Å. The structures revealed the molecular basis for α-glucosaccharide recognition by PsMBP. Overall, our findings advance the understanding of bacterial carbohydrate transport mechanisms and provide a foundation for developing efficient transport systems and new microbial biotechnological applications.

## Highlights

• A novel saccharide-binding protein (PsMBP) was identified in the *P.* FPU-7 genome
• PsMBP binds to various α-glucosaccharides, including maltose and trehalose
• Crystal structures of PsMBP complexed with various ligands were determined
• These structures reveal the molecular basis of saccharide recognition by PsMBP

## Graphical abstract

**Figure:**
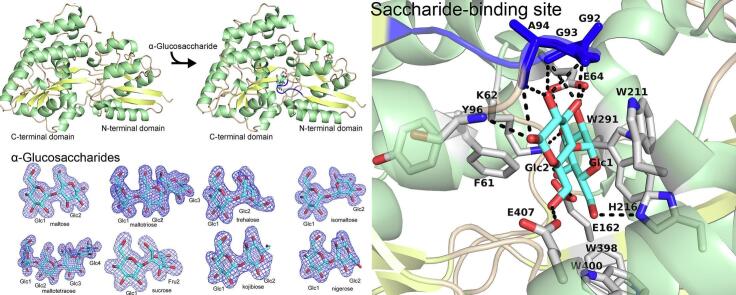

## 1 Introduction

Bacterial metabolism of carbohydrates has been extensively investigated for the effective utilization of diverse carbohydrates. Bacterial carbohydrate degradation is mediated by specific enzymes classified in the Carbohydrate-Active Enzyme database (Drula et al., 2022). Some carbohydrate-active enzymes (CAZymes) are conserved among multiple species, whereas others are species-specific. The mono-and oligosaccharides generated by these enzymes are transported into bacterial cells via two major systems: phosphotransferase (Deutscher et al., 2006) and ATP-binding cassette (ABC) transporter (Bordignon et al., 2010) systems. Each transport system exhibits distinct mechanistic features and functional advantages. The phosphotransferase system predominantly facilitates the uptake of specific monosaccharides, such as glucose, with high energy efficiency by coupling transport and simultaneous phosphorylation. In contrast, ABC transporters translocate various substrates, including carbohydrates, amino acids, ions, and other molecules (Locher, 2016). ABC importers transport not only monosaccharides and oligosaccharides but also, in certain cases, larger polysaccharides, such as alginate (Maruyama et al., 2015). Together, these two transport systems enable bacteria to efficiently assimilate diverse carbohydrates, facilitating flexible adaptations to environmental fluctuations and nutrient availability by balancing energy efficiency and substrate versatility.

Carbohydrate-specific bacterial ABC importers are classified into two subfamilies (CUT1 and CUT2) (Saier Jr., 2000). CUT1 typically contains four genes: one solute-binding protein (SBP), two transmembrane domains (TMDs), and one nucleotide-binding domain (ATPase). In contrast, CUT2 consists of three genes: SBP; TMD, which forms a homodimer; and ATPase, which is formed by the fusion of two nucleotide-binding domains (Chandravanshi et al., 2019; Schneider, 2001). ATPases harness the free energy generated by ATP hydrolysis to drive the transport of various sugars across the cell membrane, which is accompanied by conformational changes in the constituent proteins. SBPs, which are typically located in the periplasm of gram-negative bacteria or tethered to the membrane of gram-positive bacteria, play crucial roles in selectively capturing ligands and delivering them to the translocation pathway formed by TMDs, thereby determining the ligand specificity of the transport system (Maqbool et al., 2015; ter Beek et al., 2014). SBPs belong to a highly diverse protein superfamily found in both prokaryotes and eukaryotes, with over 266,000 entries in the InterPro database (Blum et al., 2025). Despite exhibiting low sequence homology, almost all SBPs share a common structural motif comprising two α/β domains connected by a flexible hinge region surrounding a central ligand-binding site (Scheepers et al., 2016).

The specific sugars transported by the ABC transporters remain unknown. Even when the ATPase, TMD, and SBP sequences indicate a sugar-importing ABC transporter, identifying the exact substrate remains challenging. In some cases, the genomic context provides important clues; genes encoding ABC transporters, along with those encoding CAZymes, are located within polysaccharide utilization loci, suggesting a functional linkage (Terrapon et al., 2018). However, this genomic colocalization is not universal; transporter genes are often spatially separated from CAZyme genes, particularly after events such as horizontal gene transfer and genomic rearrangements. Therefore, functional characterization is essential to determine the ligands of transporters with unknown ligands. This can be achieved either via genetic disruption studies, which identify the loss of specific sugar-uptake abilities, or more directly via biochemical analyses of ligand binding by SBPs or the transport activity of the full ABC system.

Previously, we isolated *Paenibacillus* sp. strain FPU-7 (*P*. FPU-7), a gram-positive bacterium known for its high chitin-degrading activity and potential industrial applications involving polysaccharides from soil (Itoh et al., 2013). This strain secretes multiple extracellular chitinases (Chi-A, −B, −C, −D, −E, −F, and −W) (Itoh et al., 2014; Itoh et al., 2013; Itoh et al., 2016). *N*,*N*′-diacetylchitobiose ((GlcNAc)_2_), the primary hydrolysis product of chitin, is captured on the cell surface by two SBPs (NagB1 and NagB2) and transported into the cell (Itoh et al., 2021), where it is hydrolyzed to GlcNAc by β-*N*-acetylglucosaminidase (Itoh et al., 2019). The chitin utilization pathway in *P.* FPU-7 has been investigated previously; however, its capacity to metabolize other carbohydrates remains unclear. Exploring its ability to utilize diverse carbohydrates along with the associated degradative enzymes and transport systems is necessary to fully leverage the biotechnological potential of *P.* FPU-7.

In this study, we functionally characterized a novel SBP from *P.* FPU-7. Biochemical analyses revealed that it binds maltose and other α-linked glucose-based oligosaccharides (α-glucosaccharides). Its amino acid sequence shares moderate similarity (approximately 25% identity) with NagB1 and NagB2, suggesting that it is an SBP. Based on their similarity to proteins with known functions, these proteins may possess sugar-binding capabilities. However, the ligand specificity remains unclear. Although the SBP was located adjacent to the two predicted TMD genes in the draft genome, no CAZyme genes were found nearby (Fig. S1). We recombinantly expressed SBP, screened for potential ligands using differential scanning fluorimetry (DSF), and confirmed its binding to α-glucosaccharides such as maltose. The dissociation constants (*K*_D_) for the bound sugars were determined using surface plasmon resonance (SPR), and the molecular basis of sugar recognition was elucidated from the crystal structures of SBP in complex with these sugars (Fig. S1).

The SBP characterized in this study are the first to add to the growing repertoire of maltose-binding proteins identified across diverse bacterial taxa (Boos and Shuman, 1998; Dahl and Manson, 1985; Hulsmann et al., 2000; Kohno et al., 2020; Schonert et al., 2006; Wassenberg et al., 2000). These proteins often vary considerably in their amino acid sequences and exhibit distinct ligand-binding affinities. Overall, the identification and functional characterization of a novel SBP with ligand-binding specificity in this study provides important insights into the diversity and functional specialization of bacterial ABC transport systems, enhancing our understanding of their roles in solute uptake and metabolic adaptation.

## 2 Materials and methods

### 2.1 Chemicals and reagents

All chemicals and reagents were of analytical grade and were purchased from FUJIFILM Wako Pure Chemical Corporation (Osaka, Japan) or Sigma-Aldrich (St. Louis, MO, USA), unless otherwise noted.

### 2.2 Amino acid sequence analysis

Primary sequence analysis was conducted using the Basic Local Alignment Search Tool (BLAST; https://blast.ncbi.nlm.nih.gov/Blast.cgi) (Altschul et al., 1990), InterPro database (https://www.ebi.ac.uk/interpro/) (Blum et al., 2025), Pfam database (http://pfam.xfam.org/) (Paysan-Lafosse et al., 2025), and Clustal Omega server (https://www.ebi.ac.uk/jdispatcher/msa/clustalo) (Madeira et al., 2024). The N-terminal signal peptide was predicted using the SignalP 6.0 server (https://services.healthtech.dtu.dk/services/SignalP-6.0/) (Nielsen et al., 2024). GPS-Palm (https://gpspalm.biocuckoo.cn/) (Ning et al., 2021) was used to predict lipid modification sites of the protein.

### 2.3 RNA extraction and quantitative polymerase chain reaction (qPCR)

*P*. FPU-7 was cultured in 2 mL of minimal medium containing 11 mM (NH_4_)_2_SO_4_, 1.7 mM NaCl, 1.4 mM KH_2_PO_4_, 3.6 mM K_2_HPO_4_, 1 mM CaCl_2_, 1 mM MgSO_4_, 50 μM FeSO_4_, 100 μM ZnSO_4_, 50 μM MnSO_4_, and 0.1 μg/mL biotin, supplemented with 5 mM maltose, trehalose, or (GlcNAc)_2_ (Tokyo Chemical Industry, Tokyo, Japan), at 28 °C with shaking. Upon reaching an optical density at 600 nm (OD_600_) of 0.5, the bacterial cells were harvested using centrifugation at 6000 ×*g* for 5 min at 4 °C.

Total RNA was extracted using a NucleoSpin RNA Purification Kit (Takara Bio, Kusatsu, Japan), and cDNA was synthesized using random hexamer priming using a ReverTra Ace qPCR RT Kit (Toyobo, Osaka, Japan). Gene expression was analyzed in triplicate using a qTOWER^3^ G thermal cycler (Analytik Jena AG, Jena, Germany) and the KAPA SYBR Fast qPCR Kit (NIPPON Genetics, Tokyo, Japan). All quantitative data were normalized to the *16S rRNA* of *P*. FPU-7. Primers used in this study are listed in Table S1.

### 2.4 Cloning of PsMBP from *P.* FPU-7

To produce recombinant PsMBP (Cys30-Lys443) with a C-terminal six-histidine tag (LEHHHHHH), *psmbp* was cloned into the pET-21b plasmid (Novagen, Madison, WI, USA). The gene was amplified from a single colony of *P*. FPU-7 using the KOD One Polymerase (Toyobo) and synthetic oligonucleotides (Table S1). Another PCR product was amplified from pET-21b using the corresponding primers (Table S1). The two PCR products were ligated using an In-Fusion HD Cloning Kit (Takara Bio). To obtain high-quality crystals, an expression vector for the truncated recombinant protein (Asn53-Lys443) was constructed using the same cloning method and specific primers (Table S1). Successful construction of plasmids was confirmed using DNA sequencing using an ABI PRISM 3130xl Genetic Analyzer (Applied Biosystems, Foster City, CA, USA).

### 2.5 Purification of recombinant PsMBP

The expression vectors were transfected into competent *Escherichia coli* BL21 (DE3) cells (Novagen). The transformants were cultured in 0.5 L of Luria–Bertani medium containing 50 μg/mL ampicillin at 37 °C. Upon reaching an OD_600_ of 0.6, protein expression was induced by adding 1.0 mM isopropyl β-D-thiogalactopyranoside, and the cultures were incubated at 20 °C for an additional 20 h. The cells were harvested using centrifugation at 6000 ×*g* for 5 min at 4 °C and lysed using ultrasonication in 10 mM sodium phosphate buffer (pH 7.4) and 0.1 mM phenylmethylsulfonyl fluoride. The cell lysate was clarified using centrifugation at 15,000 ×*g* for 20 min at 4 °C. The resulting supernatant was subjected to ammonium sulfate precipitation from 30% to 70% saturation. Precipitated proteins were dissolved in sodium phosphate buffer containing 20 mM imidazole and loaded onto a HisTrap HP Column (5 mL; Cytiva, Marlborough, MA, USA). PsMBP was eluted with a linear imidazole gradient (0.02–0.15 M) in the same buffer (50 mL), and ammonium sulfate was added to the PsMBP-containing fraction to a final concentration of 30% saturation. The sample was applied to a HiTrap Butyl HP Column (5 mL; Cytiva) pre-equilibrated with 10 mM Tris buffer (pH 7.5) containing 1.2 M (NH_4_)_2_SO_4_, and the protein was eluted using a linear gradient of (NH_4_)_2_SO_4_ (1.2–0 M) in 10 mM Tris buffer (pH 7.5; 50 mL). The eluted fractions were dialyzed overnight at 4 °C against 10 mM Tris buffer (pH 7.5). The protein concentration was determined using UV spectrophotometry and a theoretical molar extinction coefficient of 75,400 M^−1^ cm^−1^, calculated using the ExPASy ProtParam tool (http://web.expasy.org/protparam/) (Gasteiger et al., 2005). Protein purity was assessed using sodium dodecyl sulfate-polyacrylamide gel electrophoresis (SDS-PAGE), followed by CBB R-250 staining. Furthermore, molecular properties were evaluated using gel exclusion chromatography using the TSKgel SuperSW3000 Column (4.6 mm × 30 cm; Tosoh Bioscience, Tokyo, Japan) in 10 mM Tris buffer (pH 7.5) containing 0.15 M NaCl.

### 2.6 DSF

The DSF assay mixture consisted of 20 mM Tris buffer (pH 7.5), 0.25 mg/mL protein, and SYPRO Orange Protein Gel Stain (5× final concentration; Thermo Fisher Scientific, Waltham, MA, USA), with or without various saccharides (10 mM each): glucose, mannose, galactose, fructose, xylose, rhamnose, GlcNAc, cellobiose, maltose, trehalose, kojibiose, nigerose, isomaltose, sucrose, melibiose, lactulose, (GlcNAc)_2_, maltotriose, maltotetraose, α-cyclodextrin, or γ-cyclodextrin. The total reaction volume was 20 μL. Fluorescence was monitored as a function of temperature using the qTOWER^3^ G thermal cycler. The samples were heated from 25 °C to 95 °C at a constant rate of 1 °C/min. The apparent melting temperature (*T*_m,app_) was determined as the inflection point of the melting curve using the qPCRsoft ver. 4.1 (Analytik Jena AG), based on a two-state unfolding model. All assays were performed in triplicate.

### 2.7 SPR

Binding affinities of PsMBP for various α-glucosaccharides were evaluated using the Biacore X100 Plus Package (Cytiva). Briefly, PsMBP was diluted to 0.1 mg/mL in 10 mM sodium acetate buffer (pH 5.5) and covalently immobilized onto the CM5 Sensor Chip using the Amine Coupling Kit (Cytiva), achieving an immobilization level of approximately 5000 response units. Sensorgrams were recorded at 25 °C in running buffer containing 10 mM HEPES buffer (pH 7.4), 150 mM NaCl, 3 mM EDTA, and 0.005% (v/v) surfactant P20. Subsequently, binding assays were performed by injecting various concentrations of saccharide (maltose, trehalose, kojibiose, nigerose, isomaltose, sucrose, maltotriose, maltotetraose, or α-cyclodextrin) solutions over the immobilized PsMBP surface. Equilibrium *K*_D_ values were calculated by fitting the binding data to a one-site binding model (*R* = [S] *R*_max_/(*K*_D_ + [S])) using Biacore X100 evaluation software, where *R* represents the response units and [S] indicates the saccharide concentration.

### 2.8 Crystallization and X-ray diffraction

The purified protein was concentrated to 12 mg/mL using Amicon Ultra concentrators with a 10,000-molecular-weight cut-off membrane (Merck Millipore, Burlington, MA, USA). Initial crystallization screening was performed using commercial crystallization kits, Crystal Screens 1 and 2 (Hampton Research, Aliso Viejo, CA, USA) and Wizard Classic Crystallization Screens 1 and 2 (Emerald BioSystems, Bedford, MA, USA), using sitting-drop vapor-diffusion in 96-well plates at 4 or 20 °C, with and without 10 mM maltose. Crystallization conditions were subsequently optimized at 20 °C using sitting-drop vapor-diffusion in 24-well plates. Crystals were obtained from a drop (4 μL) composed of 2 μL protein solution and 2 μL reservoir solution (0.5 mL) containing 1.4 M sodium citrate. To obtain ligand-bound structures, single crystals were soaked in a solution of 5 mM Tris buffer (pH 7.5), 1.4 M sodium citrate, and either 100 mM or a saturated concentration of an α-glucosaccharide (maltose, sucrose, trehalose, isomaltose, kojibiose, nigerose, maltotriose, or maltotetraose). X-ray diffraction data were collected at the BL-26B1 station of SPring-8 (Hyogo, Japan) using the EIGER4M detector (DECTRIS Ltd., Baden-Daettwil, Switzerland) with synchrotron radiation (λ = 1.0 Å) at −173 °C under a nitrogen gas stream. The diffraction images were integrated and scaled using XDS (Kabsch, 2010) (Table S2).

### 2.9 Structure determination and refinement

The initial crystal structure of PsMBP, in the absence of a saccharide ligand, was determined using molecular replacement using the PHASER program (McCoy et al., 2007) in the PHENIX package (Liebschner et al., 2019). A search model for molecular replacement was generated using AlphaFold2 (Jumper et al., 2021) based on the amino acid sequence of PsMBP. The resulting model was rebuilt with the AutoBuild module in PHENIX and subsequently refined with Phenix.refine. To improve the model quality, iterative cycles of manual building in Coot and refinement in PHENIX were conducted (Table S2). Water molecules were automatically added using Phenix.refine and manually verified based on electron density maps; placements were accepted if the *F*_o_–*F*_c_ and 2*F*_o_–*F*_c_ maps showed densities above 3.0 and 1.0 σ, respectively. Additional water molecules were manually placed when the appropriate density was observed based on the density maps.

The crystal structure of the maltose-bound complex (PsMBP/Mal) was solved by molecular replacement in PHASER, using the ligand-free PsMBP structure as the search template. Owing to ligand-induced conformational changes, the ligand-free model was divided into two domains for separate fitting during molecular replacement. The PsMBP/Mal model was refined using the same procedure used for ligand-free structures (Table S2). Other ligand-bound structures were determined using molecular replacement using the PsMBP/Mal structure as the search model and were refined following the same protocol (Table S2).

### 2.10 Analysis of the three-dimensional structure

Structural similarities were assessed using the Protein Data Bank (PDB) (Berman et al., 2003) and the DALI server (http://ekhidna2.biocenter.helsinki.fi/dali/) (Holm et al., 2023). Structural alignments were performed via superimposition using the secondary structure-matching algorithm in Coot. Large-scale hinge-bending motions were analyzed using the DynDom web server (https://dyndom.cmp.uea.ac.uk/dyndom/) (Qi et al., 2005). Residue conservation mapped onto the three-dimensional structure was evaluated using the ConSurf server (http://consurf.tau.ac.il/) (Ashkenazy et al., 2016), which performed three iterations of Context-Specific Iterated-BLAST (Angermüller et al., 2012) and selected 150 homologous sequences (*E*-value score, 2.4 × 10^−244^ to 2.0 × 10^−69^) from the UniRef90 database (https://www.uniprot.org/uniref) (Suzek et al., 2015) for multiple sequence alignment. Finally, structural figures were generated using PyMol software (Schrödinger, New York, NY, USA).

### 2.11 Accession numbers

Nucleotide sequence data of *psmbp* were deposited in the DNA Data Bank of Japan/European Molecular Biology Laboratory/GenBank databases (https://www.ddbj.nig.ac.jp/index-e.html) under accession number LC899132. The coordinates and structure factors of the crystal structures of PsMBP (9XOR), PsMBP/Mal (9XPQ), PsMBP/Tri (9XPR), PsMBP/Tetra (9XPU), PsMBP/Suc (9XQ6), PsMBP/Tre (9XQ7), PsMBP/Iso (9XQL), PsMBP/Koji (9XRA), and PsMBP/Nige (9XRB) are available in the PDB.

## 3 Results

### 3.1 Amino acid sequence analysis of PsMBP

We searched the draft genome of *P.* FPU-7 for proteins homologous to the characterized SBPs NagB1 and NagB2 to characterize orphan SBPs with unknown saccharide ligands. Thirteen candidate sequences exhibited 20–28% identity with NagB1 or NagB2 over their entire length (>300 amino acids). Among these, we focused on one putative SBP. Two genes adjacent to this putative SBP encode TMDs homologous to those involved in saccharide transport. These TMDs shared approximately 30% identity with previously characterized TMDs such as trehalose (PDB: 7CAD) and alginate (PDB: 4TQU). Together with SBP, these TMDs may form an ABC transporter complex that is responsible for the uptake of specific saccharides. We confirmed SBP transcription in *P*. FPU-7 cells cultured in minimal medium supplemented with maltose, trehalose, or (GlcNAc)_2_. qPCR analysis detected mRNA expression under all tested conditions, with comparable levels across media; relative expression ratios were 0.67 ± 0.05 (trehalose/maltose) and 0.77 ± 0.19 ((GlcNAc)_2_/maltose) (Fig. S2). Subsequent biochemical analyses (see below) revealed that the protein exhibited a high affinity for maltose and was therefore designated as PsMBP.

InterPro and BLAST analyses predicted that PsMBP belongs to the bacterial-type extracellular SBP family. In addition to showing homology with NagB1 and NagB2, PsMBP exhibited sequence similarity to several functionally and structurally characterized SBPs, including the raffinose/panose-binding protein BlG16B from *Bifidobacterium animalis* subsp. *lactis* Bl-04 (28% identity) (Ejby et al., 2016), manno-oligosaccharide-binding protein CpMnBP1 from *Caldanaerobius polysaccharolyticus* strain ATCC BAA-17 (27% identity) (Chekan et al., 2014), and xylo-oligosaccharide-binding protein XBP1 from the same strain (25% identity) (Han et al., 2012). Owing to the diversity of saccharide ligands recognized by these homologous proteins, the specific ligand(s) of PsMBP could not be predicted based solely on sequence similarity.

A signal peptide was predicted at the N-terminus (Met1-Gly29), and a lipid modification site was identified at Cys30. Consistent with the lipoproteins of gram-positive bacteria (Babu et al., 2006), PsMBP may be anchored to the plasma membrane via a lipid moiety bound to the conserved lipobox motif, Val27-Cys30 (VAGC).

### 3.2 Affinity of PsMBP for α-glucosaccharides

A recombinant protein lacking the predicted N-terminal signaling peptide was heterologously expressed in *E. coli* to investigate the ligand-binding properties of PsMBP. Approximately 150 mg of purified protein was obtained from 3 g of wet *E. coli*. The molecular mass estimated from the migration distance in SDS-PAGE was approximately 43 kDa, which is lower than the theoretical mass of 47 kDa calculated from the amino acid sequence. Gel filtration chromatography indicated that PsMBP existed predominantly as a monomer in solution; however, its apparent molecular mass was lower than the theoretical value of approximately 47 kDa (Fig. S3). A truncated version of PsMBP was generated to facilitate crystallization. Based on the sequence alignment with NagB1 and NagB2 (Fig. S4), the N-terminal residues (Cys30-Lys52) of PsMBP were removed. These residues correspond to regions in NagB1 and NagB2 that have not been resolved in their crystal structures.

**Fig. 1:**
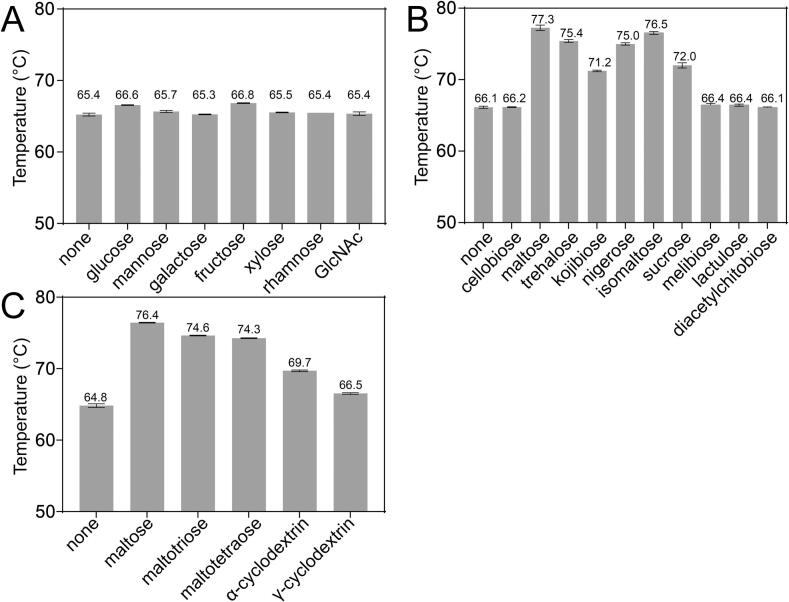
Differential scanning fluorimetry (DSF) analysis of a novel solute-binding protein (SBP) from *Paenibacillus* sp. str. FPU-7 (PsMBP) and various saccharides. The apparent melting temperature (*T*_m,app_) values were determined as the inflection points of PsMBP melting curves in the presence of various saccharides (10 mM each). (A) None of the tested monosaccharides (glucose, mannose, galactose, fructose, xylose, rhamnose, and GlcNAc) significantly increased the *T*_m,app_ values. (B) The *T*_m,app_ values increased when mixed with α-gluco-di-saccharides, such as maltose, trehalose, kojibiose, nigerose, isomaltose, and sucrose, but not with cellobiose, melibiose, lactulose, and *N*,*N*′-diacetylchitobiose ((GlcNAc)_2_). (C) Addition of α-gluco-oligosaccharides, such as maltotriose, maltotetraose, and α-cyclodextrin, also increased the *T*_m,app_ values.

The PsMBP ligand was initially screened using DSF using various mono- and disaccharides (Figs. 1A, B, and S5; Table S3). The addition of 10 mM α-glucodisaccharides (disaccharides consisting of glucose at the non-reducing end and linked via an α-glycosidic bond), such as maltose, trehalose, kojibiose, nigerose, isomaltose, and sucrose, caused significant thermal shifts (Δ*T*_m,app_) of 5.1–11.2 °C. In contrast, other tested saccharides, including various monosaccharides, yielded Δ*T*_m,app_ values comparable to those of the no-ligand control, indicating negligible binding. No significant thermal shift was detected for cellobiose (Glc-Glc, β-(1 → 4)), which contains a β-glycosidic linkage, or melibiose (Gal-Glc, α-(1 → 6)), which, despite exhibiting an α-linkage, features galactose at the non-reducing end. Notably, saccharides with a degree of polymerization greater than two, such as maltotriose and maltotetraose, also showed increased *T*_m,app_ values. Additionally, a measurable thermal shift was observed for α-cyclodextrin (Δ*T*_m,app_ = 4.9 °C; Fig. 1C). To further characterize binding affinities, equilibrium *K*_D_ values for these α-glucosaccharides were determined using SPR analysis (Table 1; Fig. S6). The *K*_D_ values ranged from the nanomolar to the micromolar range, consistent with those reported for other sugar-binding SBPs (Berntsson et al., 2010). Among the tested ligands, maltose exhibited the highest binding affinity for PsMBP with a *K*_D_ of 31.5 nM (Table 1).

**Table 1:** Dissociation constants of PsMBP and oligosaccharides.

Saccharide	*K*_D_ (nM)	χ^2^^a^	Glycosidic bond
Maltose	31.5 ± 3.9	0.107	α-(1 → 4)
Trehalose	47.0 ± 4.4	0.114	α-(1 → 1)
Kojibiose	4500 ± 430	0.105	α-(1 → 2)
Nigerose	N.D.^b^		α-(1 → 3)
Isomaltose	88.4 ± 6.5	0.232	α-(1 → 6)
Sucrose (Glc-Frc)	321 ± 73	0.654	α-(1 → 2)
Maltotriose	196 ± 19	0.156	α-(1 → 4)
Maltotetraose	175 ± 12	0.183	α-(1 → 4)
α-Cyclodextrin	4700 ± 600	0.151	α-(1 → 4)

a Averaged squared residual per data point (χ^2^ = Σ (*y*_i_ – *ŷ*_i_)^2^/(*n* – *p*)), where *y*_i_ is the fitted value at each data point, *ŷ*_i_ is the experimental value at the point, *n* is the number of data points, and *p* is the number of fitted parameters.

b Based on the sensorgrams, binding of nigerose to PsMBP was confirmed; however, the dissociation constant (*K*_D_) could not be determined due to large variations in response units (RUs).

### 3.3 Overall structure of PsMBP

**Fig. 2:**
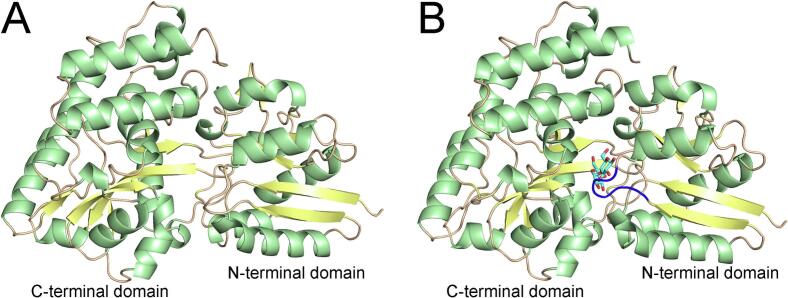
Overall structure of PsMBP. Ribbon diagrams of PsMBP in the ligand-free (A) and maltose-bound (PsMBP/Mal) (B) forms are shown. The protein adopts a typical SBP fold, comprising two globular α/β domains: an N-terminal domain and a C-terminal domain. In the PsMBP/Mal structure, a maltose molecule (cyan stick model) is bound at the interdomain ligand-binding cleft. The loop region spanning residues Ser89-Ala94 (S89–A94 loop) is highlighted in blue. (For interpretation of the references to colour in this figure legend, the reader is referred to the web version of this article.)

X-ray crystallographic analysis was performed to assess the mechanistic recognition of α-glucosaccharides by PsMBP (Fig. 2A; Table S2). Under the crystallization conditions tested, only crystals of the ligand-free form were obtained with no ligand co-crystals. Therefore, crystal structures of PsMBP in complex with eight different saccharides, maltose, trehalose, isomaltose, sucrose, maltotriose, maltotetraose, kojibiose, and nigerose, were obtained by soaking the respective ligands into crystals, yielding resolutions of 1.4–2.1 Å (Fig. 2B; Figs. S7 and S8, Table S2). The detailed structural features of the ligand-binding sites are described below. The overall structure adopted a typical SBP fold (Berntsson et al., 2010) consisting of two globular α/β domains, similar to those of high-sequence-homology proteins such as NagB1 and NagB2 of *P*. FPU-7 (Itoh et al., 2021). The N-terminal domain (residues Asn52-Asn160 and Val326-Asn393) comprised six β-strands and seven α-helices, whereas the C-terminal domain (residues Phe166-Lys321 and Asn399-Lys443) contained five β-strands and 12 α-helices. These two domains were connected by three antiparallel β-strands formed by residues Leu161-Gly165, Leu322-Gly325, and Lys394-Trp398. The asymmetric unit of the crystal contains two polypeptide chains (A and B). Chain A, which exhibited a lower average *B*-factor than chain B, was selected as the representative model for the subsequent analyses. A minor difference in interdomain closure was observed between the two chains, with a rotation angle of 3.2°, as determined using DynDom (Qi et al., 2005), with chain A adopting a slightly more closed conformation. The Cα root-mean-square deviation (RMSD) between chains A and B was 0.503 Å.

### 3.4 Ligand-binding sites of PsMBP

**Fig. 3:**
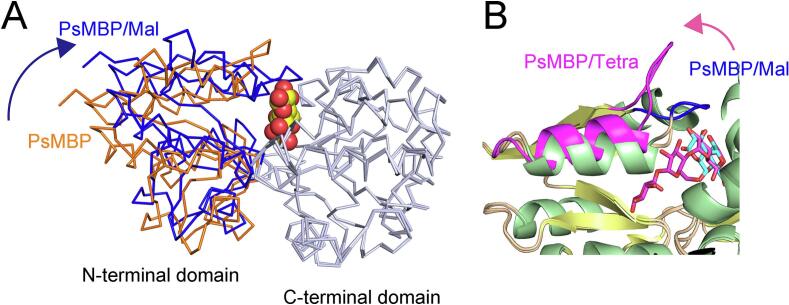
Domain motion during ligand binding (A) and conformational differences in the S89–A94 loop (B). (A) Upon ligand binding, PsMBP underwent a conformational change via rigid-body rotation, consistent with the general mechanism observed in other SBPs. Superposition of the C-terminal domains (gray) of the ligand-free and maltose-bound (PsMBP/Mal) structures revealed that the N-terminal domain of PsMBP/Mal (blue) rotated by 14.1° toward the ligand-binding site relative to the unbound form (PsMBP; orange). (B) The S89–A94 loop in chain A adopted a distinct conformation in PsMBP/Tri, PsMBP/Tetra, and PsMBP/Iso compared to that in other ligand-bound forms. Superposition of chain A from PsMBP/Tetra and PsMBP/Mal revealed that, in PsMBP/Tetra, the loop (magenta) was displaced, along with the adjacent α-helix, in the open conformation. In contrast, in other ligand-bound structures, such as PsMBP/Mal (blue), the loop remained close to the ligand. (For interpretation of the references to colour in this figure legend, the reader is referred to the web version of this article.)

A comparison of the ligand-bound and unbound crystal structures revealed that ligand binding induced domain closure through a rigid-body rotation of 9.9–14.5° around the hinge region (Fig. 3A). Although the angles were possibly influenced by crystal packing, such large hinge-bending motions were consistent with those reported for other SBPs of type I ABC importers (Lewinson and Livnat-Levanon, 2017; Sharff et al., 1992). All the ligand-bound structures exhibited closed conformations; however, local conformational differences were also observed. Specifically, domain closure was more pronounced in the structures bound to maltose (14.1°), trehalose (14.5°), and sucrose (14.5°) than in those bound to maltotriose (11.5°), maltotetraose (11.3°), isomaltose (11.1°), kojibiose (9.9°), and nigerose (11.3°). Additionally, in the structures of PsMBP/maltotriose (Tri), PsMBP/maltotetraose (Tetra), PsMBP/isomaltose (Iso), PsMBP/kojibiose (Koji), and PsMBP/nigerose (Nige), the conformation of a loop spanning Ser89-Ala94 (sequence: SVGGGA), hereafter referred to as the S89–A94 loop, differed between the two chains (A and B) in the asymmetric unit. In chain B of these five structures, the S89–A94 loop is positioned toward the ligand-binding site, forming a closed conformation. In contrast, in chain A, the loop was lifted away from the α-helix to which it was connected, adopting an open conformation (Fig. 3B). In other ligand-bound structures (PsMBP/Mal, PsMBP/sucrose (Suc), and PsMBP/trehalose (Tre)), the S89–A94 loop consistently adopted a closed conformation in both chains (Fig. 3B). The average *B*-factor of the entire polypeptide chain in PsMBP was 25.1 Å^2^, whereas the S89–A94 loop exhibited a higher average *B*-factor of 33.3 Å^2^. Notably, the central region of this loop containing three consecutive glycine residues, showed greater flexibility, with an average *B*-factor of 37.4 Å^2^. Collectively, these results suggest that the S89–A94 loop (Fig. 3B) is structurally flexible and plays a role in accommodating ligands of different sizes or conformations.

**Fig. 4:**
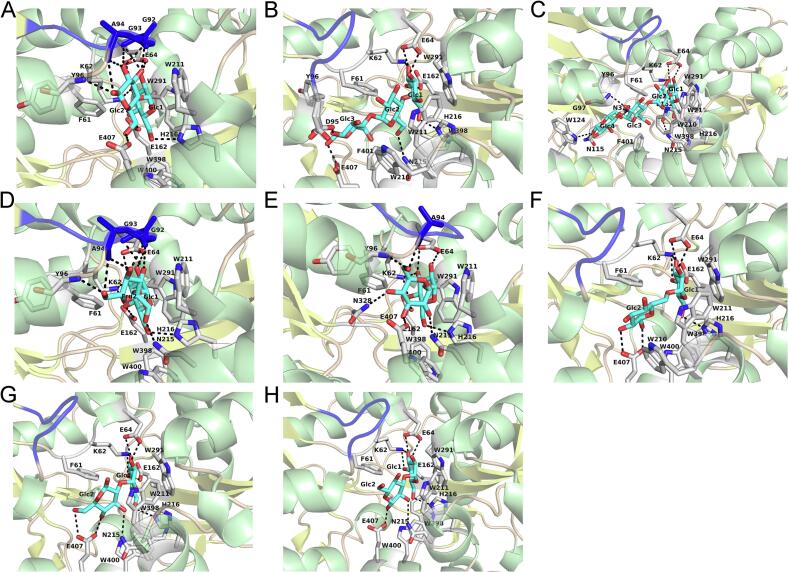
Ligand-binding sites of PsMBP in the following complexes: PsMBP/Mal (A), PsMBP/Tri (B), PsMBP/Tetra (C), PsMBP/Suc (D), PsMBP/Tre (E), PsMBP/Iso (F), PsMBP/Koji (G), and PsMBP/Nige (H). Amino acid residues involved in ligand interactions are shown as gray stick models, and the bound saccharides are shown as cyan stick models. Hydrogen bonds are indicated by black dashed lines, with interatomic distances ≤3.5 Å. The structure of the S89–A94 loop is represented by the blue ribbon model. (For interpretation of the references to colour in this figure legend, the reader is referred to the web version of this article.)

The interactions between PsMBP and bound saccharides are illustrated in Fig. 4 and summarized in Table S4. The saccharide units were numbered starting from the non-reducing end (position 1), and this nomenclature was applied consistently across all ligands. The orientation and binding modes of the first glucose residue (Glc1) are conserved among all ligand-bound structures. Glc1 was stacked against Trp291 and engaged in CH/π interactions with Trp291. In addition, it was found to be in contact with Phe61, Trp398, and Trp400. Hydrogen bonds were observed between Glc1 and several residues; O2 and O3 interacted with Lys62, O4 interacted with Glu162, and O6 interacted with His216 and Glu64. In contrast, the interactions involving the second saccharide unit (Glc2 or Fru2) varied depending on the ligand. In PsMBP/Mal (Fig. 4A), PsMBP/Tri (Fig. 4B), PsMBP/Tetra (Fig. 4C), PsMBP/Tre (Fig. 4E), PsMBP/Koji (Fig. 4G), and PsMBP/Nige (Fig. 4H), Glc2 was stacked on Trp211, engaging in CH/π interactions. In PsMBP/Mal, Glc2 forms hydrogen bonds with the backbone atoms of the S89–A94 loop (O1, O2, O3, and O6) and Glu407 via an O2 atom. However, in PsMBP/Tri and PsMBP/Tetra, the O2 atom of Glc2 interacts with Asn215 instead of Glu407. The Fru2 moiety of PsMBP/Suc (Fig. 4D), Glc2 of PsMBP/Tre (Fig. 4E), Glc2 of PsMBP/Koji (Fig. 4G), and Glc2 of PsMBP/Nige (Fig. 4H) occupied positions similar to Glc2 in PsMBP/Mal (Fig. 4A); however, their hydrogen bonding patterns differed. In PsMBP/Iso (Fig. 4F), Glc2 was not aligned parallel to the indole ring of Trp211 and was displaced due to the difference between the α-(1 → 6)- and α-(1 → 4)-linkages. The Glc3 residues in PsMBP/Tri (Fig. 4B) and PsMBP/Tetra (Fig. 4C) interacted with Tyr96, Trp210, and Phe401, respectively. In PsMBP/Tri, Glc3 formed hydrogen bonds through the O1 and O2 atoms with Glu407 and Asp95, respectively. However, in PsMBP/Tetra, these residues were slightly farther apart and did not form hydrogen bonds. The fourth glucose unit (Glc4) in PsMBP/Tetra (Fig. 4C) interacted with Tyr96 and Phe401 to form several hydrogen bonds: O1 with Asn115 and Trp124, O2 with Trp124, and O6 with Asn328.

### 3.5 Structural similarity

**Fig. 5:**
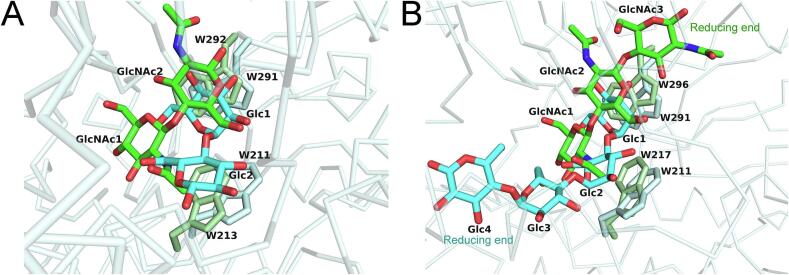
Structural comparisons of PsMBP with NagB1 and NagB2. (A) The structure of PsMBP bound to maltose (PsMBP/Mal; light blue ribbon) was superimposed onto that of NagB1 bound to (GlcNAc)₂ (light green ribbon). The ligands are shown as stick models, with maltose in cyan and (GlcNAc)_2_ in green. The saccharide units are numbered 1 and 2, starting from the non-reducing end. Both disaccharides were bound in similar positions, but the sugar order was reversed: Glc1 occupied the same position as GlcNAc2, whereas Glc2 was located at the *N*-acetyl group of GlcNAc1. (B) The structure of PsMBP bound to maltotetraose (PsMBP/Tetra; light blue ribbon) was superimposed onto that of NagB2 bound to (GlcNAc)_3_ (light green ribbon). The ligands are shown as stick models, with maltotetraose in cyan and (GlcNAc)_3_ in green. The saccharide units are numbered 1 to 4 from the non-reducing end. In both comparisons, the proteins were bound to oligosaccharides at similar positions; however, the orientation of the sugar chains was reversed, with the reducing-end sugars facing opposite solvent sides. (For interpretation of the references to colour in this figure legend, the reader is referred to the web version of this article.)

**Fig. 6:**
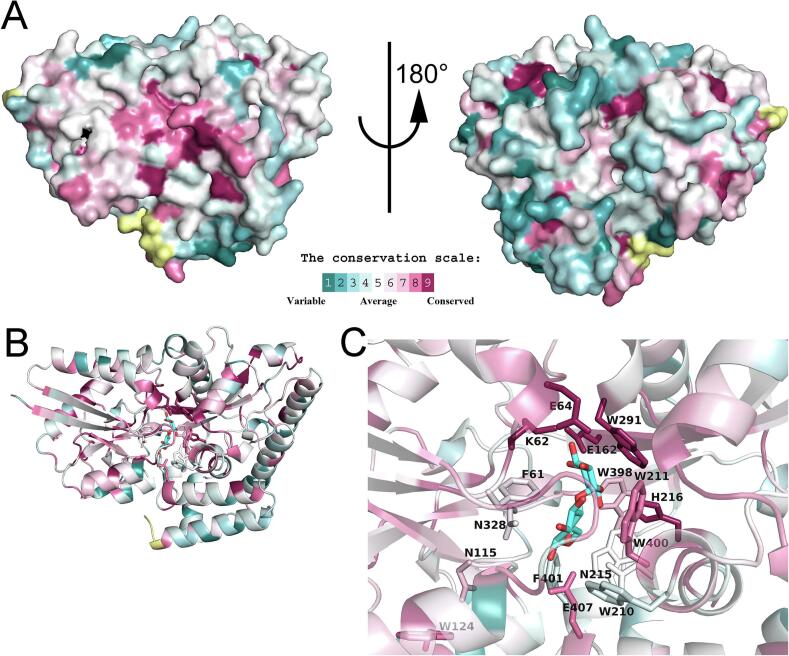
Amino acid conservation mapped onto the PsMBP structure. (A) Surface representation of PsMBP colored according to sequence conservation analyzed using the ConSurf server: Variable regions in cyan, moderately conserved regions in white, and highly conserved regions in magenta. (B) Ribbon representation of PsMBP with the same conservation coloring scheme to visualize the conserved regions in the structural fold. Bound maltose is shown as a cyan stick model. (C) Close-up view of the ligand-binding site highlighting the conserved residues involved in saccharide recognition. (For interpretation of the references to colour in this figure legend, the reader is referred to the web version of this article.)

DALI server analysis revealed that the overall structural fold of PsMBP exhibited notable similarity to that of NagB1 (PDB: 7EHP), with a *Z*-score of 38.1, an RMSD of 2.5 Å over 397 Cα atoms, and a sequence identity of 22%. Similarly, PsMBP resembled NagB2 (PDB: 7EHQ), with a Z-score of 38.8, an RMSD of 2.7 Å over 406 Cα atoms, and a sequence identity of 25%. Structural similarities were also observed with many other SBPs recognizing oligosaccharides, including a xylo-oligosaccharide-binding protein from *Bifidobacterium* (PDB: 3ZKK; Z-score: 37.3; RMSD: 2.6 Å over 371 Cα atoms; 22% sequence identity) (Ejby et al., 2013), raffinose/panose-binding protein from *Bifidobacterium* (PDB: 4ZS9; Z-score: 37.2; RMSD: 2.4 Å over 361 Cα atoms; 23% sequence identity) (Ejby et al., 2016), and trehalose/maltose-binding protein from *Thermococcus* (PDB: 1EU8; Z-score: 33.7; RMSD: 2.7 Å over 365 Cα atoms; 16% sequence identity) (Diez et al., 2001). Although the overall structures of these proteins are similar, their ligand-binding sites differ significantly (Fig. S9). For example, a comparison of PsMBP with NagB1 or NagB2 (Fig. 5) revealed that only Trp291 and Trp211, which interact with Glc1 and Glc2, respectively, in PsMBP/Mal, occupied conserved positions in the ligand-binding pocket. The saccharide units interacting with Trp291 (in PsMBP), Trp292 (in NagB1), or Trp296 (in NagB2) were located at similar locations across all structures (Fig. 5); however, the adjacent saccharide units (Glc2 in PsMBP or GlcNAc1 in NagB1/NagB2) were positioned differently. Notably, Trp213 (NagB1; Fig. 5A) and Trp217 (NagB2; Fig. 5B), which correspond to PsMBP Trp211, interacted with the *N*-acetyl group of GlcNAc1, rather than with the sugar ring (Fig. 5). Furthermore, a comparison between PsMBP/Tetra and NagB2/(GlcNAc)_3_ (Fig. 5B) indicated that although both proteins bound to the non-reducing-terminal saccharide deep within the pocket and oriented the reducing terminal toward the solvent-exposed surface, the directionality of the saccharide-binding sites was reversed. The positions of the non-reducing terminal saccharides also differed between the two structures. Although the sugar chains were oriented in opposite directions, the spatial positioning of the α-(1 → 4) and β-(1 → 4) linkages within the binding cleft was conserved despite their stereochemical differences. Specifically, the position of the α-(1 → 4) glycosidic bond between Glc1 and Glc2 in PsMBP corresponds to that of the β-(1 → 4) bond between GlcNAc1 and GlcNAc2 in NagB1 (or NagB2) (Figs. 6, S10A, B). In other SBPs sharing the same three-dimensional structure as PsMBP, the sugar chains were similarly positioned along the cleft between the two domains (Fig. S9A). Although their orientations varied, the positions of the glycosidic bonds were conserved (Fig. S9B).

### 3.6 Conservation of structure and amino acid sequence

The structural and sequence conservation of PsMBP and related functionally uncharacterized proteins were visualized using the ConSurf server (Fig. 6). Protein sequences for multiple sequence alignment were randomly selected from uncharacterized SBPs of gram-positive bacteria, including *Paenibacillus*, *Halobacillus*, *Fictibacillus*, *Cohnella*, *Ectobacillus*, and *Saccharibacillus*, with a sequence identity of 40–90%. Among the residues involved in hydrogen bonding with saccharides in PsMBP, Lys62, Glu64, Glu162, His216, and Glu407 are highly conserved. In contrast, Asn215 and Asn115 were not conserved. Two tryptophan residues (Trp291 and Trp211), which participated in CH/π stacking with the sugar ring, along with the S89–A94 loop involved in local conformational changes, were conserved except for Ala94. The residues contributing to the hydrophobic surface of the binding site (Phe61, Trp400, Trp210, and Phe401) were not conserved. Based on the structures of other ABC transporters (Thomas and Tampe, 2020) and their transport mechanisms, ligand binding is likely to occur through closed surfaces. The surface formed upon the two domains closing after saccharide binding showed higher amino acid conservation than the opposite surface (Fig. 6).

## 4 Discussion

*P*. FPU-7 efficiently degrades chitin into (GlcNAc)_2_, which acts as a carbon and nitrogen source (Itoh et al., 2013). In bacteria, oligosaccharides are transported through various transport systems (Jeckelmann and Erni, 2020). Bacterial ABC transporters typically consist of SBP, TMD(s), and ATPase, with SBP determining specificity by binding to the ligand (Maqbool et al., 2015). We previously identified two homologous SBPs (NagB1 and NagB2) from *P.* FPU-7 that specifically bind to (GlcNAc)_2_ on the cell surface, indicating their role in the recognition and uptake of this disaccharide (Itoh et al., 2021). In the present study, we performed a homology search based on the amino acid sequences of NagB1 and NagB2 to identify additional SBPs potentially involved in carbohydrate binding in *P.* FPU-7. Our search identified several candidate genes. qPCR analysis demonstrated that the gene encoding PsMBP was expressed in *P.* FPU-7 cells cultured under standard conditions. Sequence analysis of PsMBP revealed features characteristic of oligosaccharide-binding SBPs; however, the specific ligand remains unknown. Therefore, we conducted biochemical and structural analyses to elucidate the ligand specificity and recognition mechanisms.

We found that PsMBP was an SBP with α-glucosaccharide ligands, such as maltose and trehalose, as indicated using DSF (Fig. 1) and SPR analysis (Table 1). DSF studies were conducted with ligands at concentrations of up to 10 mM under conditions expected to allow significant binding to PsMBP. The Δ*T*_m,app_ values correlated with the *K*_D_ values, indicating that ligands with higher affinities resulted in larger Δ*T*_m,ap_ values. A similar trend—where higher Δ*T*_m,app_ values correlate with lower *K*_D_ values—has also been observed in maltodextrin-binding SBPs from *Anaerocellum bescii* (although *T*_m_ values were determined by differential scanning fluorimetry and *K*_D_ values by isothermal titration calorimetry) (Tjo et al., 2025).

PsMBP bound to maltose. However, its amino acid sequence shares low identity (< 23%) with well-characterized maltose-binding proteins from *E. coli* (MalE), *Bacillus subtilis* (MdxE), *Thermotoga maritima* (TmMBP), and *Thermococcus litoralis* (TlMBP). In the draft genome of *P.* FPU-7, no sequence showed more than 30% identity with any known maltose-binding protein. The *K*_D_ for maltose binding to MalE is approximately 1–3 μM (Bao and Duong, 2012; Hall et al., 1997; Jacso et al., 2012; Telmer and Shilton, 2003). MdxE exhibits a *K*_D_ of 1 mM for maltose and binds to maltodextrins with higher affinity (*K*_D_ = 3 μM) (Schonert et al., 2006). TmMBP binds to maltose with a *K*_D_ of 7.2 μM (Wassenberg et al., 2000). In contrast, PsMBP exhibited a *K*_D_ of 31.5 nM for maltose, indicating a stronger binding affinity than MalE, MdxE, and TmMBP. Furthermore, PsMBP, but not MalE, can bind sucrose (*K*_D_ = 320 nM) (Hall et al., 1997). TlMBP, which binds to both maltose and trehalose, has *K*_D_ values similar to those of PsMBP (160 nM for maltose and trehalose) (Horlacher et al., 1998). These differences reflect variations in amino acid sequence identity and ligand-binding sites and highlight the distinct saccharide-binding properties of these proteins. MalE is widely used as a tag for protein expression and purification (Riggs, 1997). Our newly characterized PsMBP could also be used as a protein tag with novel selectivity owing to its unique affinity for maltose, trehalose, and sucrose.

The newly identified PsMBP specifically bound to α-glucosaccharides, such as maltose, but not to cellobiose (a disaccharide composed of β-linked glucose units). It also recognized disaccharides in which the second sugar was fructose or glucose and accommodated linkages other than α-(1 → 4), indicating relatively broad specificity. The highest affinity was observed for maltose and trehalose. X-ray crystallographic analysis of PsMBP was performed to elucidate the structural basis for this specificity. The overall structure of PsMBP closely resembles that of other SBPs, including the xylo-oligosaccharide-binding protein from *Bifidobacterium* (Ejby et al., 2013), the raffinose/panose-binding protein from *Bifidobacterium* (Ejby et al., 2016), the trehalose/maltose-binding protein from *Thermococcus* (Diez et al., 2001), and NagB1 and NagB2. However, consistent with their low amino acid sequence identity, the arrangement of residues in the ligand-binding sites varied substantially among these proteins, leading to significant differences in the positions of bound ligands. In PsMBP, the non-reducing terminal glucose (Glc1) was tightly bound at the base of the interdomain pocket via an extensive network of hydrogen bonds and CH/π interactions. In contrast, the remaining α-linked saccharide residues extended outward toward the solvent. In contrast, in NagB1 and NagB2, the SBPs that recognized β-linked chitin oligosaccharides and shared the highest sequence similarity with PsMBP among the structurally characterized proteins, the direction of oligosaccharide binding was reversed (Fig. 5). Although two highly conserved tryptophan residues involved in sugar stacking occupied similar positions in PsMBP, NagB1, and NagB2, the alignment of the bound saccharides differed, with Glc1 in PsMBP corresponding to GlcNAc2, rather than GlcNAc1 in NagB1/NagB2. The spatial positioning of the glycosidic bond between the non-reducing end saccharide and the adjacent saccharide was identical in PsMBP and NagB1 (NagB2). The glycosidic oxygen atom was oriented toward Phe61 of PsMBP. This phenylalanine residue is conserved in NagB1 (Phe115) and NagB2 (Phe116), along with a tryptophan residue involved in CH/π interactions (Figs. S10A, B). Although this phenylalanine residue was absent from the xylo-oligosaccharide-binding protein from *Bifidobacterium animalis* (Fig. S10C), it was retained in a raffinose/panose-binding protein from the same organism (Phe57) (Fig. S10D) and maltose-binding protein from *Thermotoga maritima* (TmMBP) (Phe41) (Fig. S10E). In the trehalose/maltose-binding protein from *Thermococcus litoralis* (TlMBP), a tryptophan residue (Trp295) occupies the corresponding position (Fig. S10F).

Residues surrounding the non-reducing terminal glucose (Glc1), including Trp291, Lys62, Glu64, Glu162, and His216, were highly conserved and represented distinctive features of PsMBP among α-glucosaccharide-binding proteins, distinguishing it from other saccharide-binding MBPs (Fig. 6). In contrast, the conservation of amino acid residues progressively decreased at the binding sites of the second and third saccharide units, which is consistent with the broader substrate specificity observed at these positions. For example, PsMBP accommodated fructose instead of glucose as the second saccharide and recognized glucose linked via α-(1 → 1), α-(1 → 2), α-(1 → 3), α-(1 → 4), or α-(1 → 6) bond. The second and subsequent saccharide units were possibly recognized through spatial accommodation involving weak interactions and the flexible rearrangement of sugar positions and hydrogen bonding networks. Structural analysis revealed two distinct conformations of the S89–A94 loop that interacted with the second saccharide. All residues in this loop, except Ala94, are highly conserved and specific to the PsMBP family, suggesting that loop mobility plays a critical role in ligand affinity. Notably, ligand binding induces domain closure, the extent of which varies depending on the ligand. In the PsMBP/Mal structure, in which the loop adopts a closed conformation, the two domains rotate by 14.1° relative to the ligand-free structure. In contrast, in the chain A structures of PsMBP bound to maltotetraose, maltotriose, isomaltose, kojibiose, and nigerose, domain closure was less pronounced (e.g., 11.3° for PsMBP/Tetra and 9.9° for PsMBP/Koji), corresponding to a difference of approximately 3–4°. Interestingly, *K*_D_ values for maltotriose (196 nM), maltotetraose (175 nM), isomaltose (88.4 nM), and kojibiose (4.5 μM) were higher than that for maltose (31.5 nM) despite sufficient binding capacity for these ligands (Fig. 4). A similar trend was observed in the thermal shift assay, where Δ*T*_m,app_ was the highest for maltose (Fig. 1). These findings suggest that the degree of domain closure and S89–A94 loop conformation contribute to ligand affinity. However, in the case of sucrose (Glc–Fru), despite the loop adopting the closed conformation, *K*_D_ (321 nM) was approximately 10-fold higher than that of maltose. These differences in *K*_D_ values among the various ligands against PsMBP suggest the involvement of additional factors. Differences in the number of hydrogen bonds with the second sugar may have contributed to the differences in affinity. For example, Fru2 in PsMBP/Suc formed seven hydrogen bonds, fewer than the eight hydrogen bonds formed by Glc2 in PsMBP/Mal but more than those observed in PsMBP/Tre (five), PsMBP/Iso (two), PsMBP/Koji (three), and PsMBP/Nige (two). Additionally, entropy effects mediated by water molecules may also contribute to differences in binding affinity. Eleven water molecules were bound to the binding site of PsMBP/Iso, higher than the number bound to the binding sites of PsMBP/Mal (four), PsMBP/Suc (four), PsMBP/Tre (five), PsMBP/Koji (four), and PsMBP/Nige (four). This might have contributed to the larger *K*_D_ observed for isomaltose than for maltose.

*P*. FPU-7 was grown with maltose, trehalose, and sucrose as the sole carbon sources. The presence of a gene encoding β-amylase in the draft genome suggests the potential capacity of this bacterium to hydrolyze and utilize starch. Although PsMBP binds to maltotriose and maltotetraose, it exhibits the highest binding affinity for maltose. This finding implies that PsMBP plays a pivotal role in capturing maltose generated using β-amylase activity, facilitating its transport into the cell. Once imported, maltose may be metabolized by intracellular enzymes such as α-glucosidase or maltose phosphorylase. Trehalose exhibited the second-highest binding affinity for PsMBP, with a *K*_D_ of 47 nM, suggesting that PsMBP also contributes to trehalose uptake. Because trehalose is commonly found in fungi and insects, *P.* FPU-7 may exploit this disaccharide, in addition to chitin, as a nutrient source in the soil. Although starch metabolism has been extensively characterized in model organisms, such as *E. coli* and *B. subtilis*, further investigations are necessary to clarify the starch-degrading capacity of soil-dwelling *Paenibacillus* species, including strain FPU-7, which may possess starch uptake pathways distinct from those of well-studied model organisms.

In conclusion, this study identified an uncharacterized SBP, a maltose-binding protein from *Paenibacillus* sp., and elucidated its binding specificity for α-glucosaccharides using crystal structures. Our findings suggest that bacteria have evolved highly specialized SBPs tailored to their ecological niches and nutrient availability. Furthermore, our results suggest that many SBPs remain functionally uncharacterized because their ligand specificity cannot be reliably predicted solely from amino acid sequence homology.

## CRediT authorship contribution statement

**Takafumi Itoh:** Writing – original draft, Visualization, Validation, Supervision, Project administration, Methodology, Investigation, Funding acquisition, Formal analysis. **Kanato Kataoka:** Writing – review & editing, Investigation. **Yuma Kaneko:** Writing – review & editing, Investigation. **Takao Hibi:** Writing – review & editing, Validation, Supervision, Investigation, Formal analysis. **Hisashi Kimoto:** Writing – review & editing, Supervision, Resources, Project administration, Conceptualization.

## Funding

This study was partially supported by a Grant-in-Aid for Scientific Research (C) [grant number 23K05494 to T.I.] from the 10.13039/501100001691Japan Society for the Promotion of Science.

## Declaration of competing interest

The authors declare the following financial interests/personal relationships which may be considered as potential competing interests:

Takafumi Itoh reports financial support was provided by The Japan Society for the Promotion of Science. If there are other authors, they declare that they have no known competing financial interests or personal relationships that could have appeared to influence the work reported in this paper.

## Data Availability

Data will be made available on request.
