# Supplementary material for: Structural characterization of an α-glucosaccharide-binding protein from Paenibacillus sp. str. FPU-7
Source: J Struct Biol X. 2026 May 28;14:100149. doi: 10.1016/j.yjsbx.2026.100149 (PMC13311272; doi:10.1016/j.yjsbx.2026.100149)
Supplement: Supplementary file 1 — Supplementary material [file mmc1.pdf]

Supplementary Information

Structural characterization of an α-glucosaccharide-binding protein from *Paenibacillus* sp. str. FPU-7

Takafumi Itoh[a,*], Kanato Kataoka[a], Yuma Kaneko[a], Takao Hibi[a], and Hisashi Kimoto[a,*]

[a]Department of Bioscience and Biotechnology, Fukui Prefectural University, 4-1-1, Matsuokakenjyojima, Eiheiji-cho, Yoshida-gun, Fukui, 910-1195, Japan

***Corresponding authors:** Takafumi Itoh and Hisashi Kimoto, Department of Bioscience and Biotechnology, Fukui Prefectural University, 4-1-1 Matsuokakenjyoujima, Eiheiji-cho, Yoshida-gun, Fukui 910-1195, Japan; Phone +81-776-61-6000; Fax +81-776-61-6015; E-mail ito-t@g.fpu.ac.jp, kimoto@g.fpu.ac.jp

Table S1. Oligonucleotide primers used in this study.

Primer name	Primer sequence (5′ to 3′)
qPCR	
PsMBP_qPCR-F	AATGGCAGGAGCATCTGGAG
PsMBP_qPCR-F	AATGGCAGGAGCATCTGGAG
PsFPU7_16SrRNA-F	ATGCGTAGCCGACCTGAGA
PsFPU7_16SrRNA-R	GCGTTCTTCCTTGGCAACAG
Cloning	
PsMBP_Cys30-F	GGAGATATA**CATATG**TGCGCCAATAAAACAGAAACGCC
PsMBP_Asn53-F	GGAGATATA**CATATG**AACGTTACACTGAAGATGTTTCAAT
PsMBP-R	GTGGTGGTG**CTCGAG**TTTTTTCAGGCTATCCCAAGTTTG
pET21b-F	**CTCGAG**CACCACCACCACCACCACTGAGAT
pET21b-R	**CATATG**TATATCTCCTTCTTAAAG

Bold characters indicate recognition site of restriction enzyme, NdeI or XhoI.

Table S2. Data collection and refinement statistics for PsMBP crystal structures.

	**PsMBP**	**PsMBP/Mal**	**PsMBP/Tri**	**PsMBP/Tetra**	**PsMBP/Suc**	**PsMBP/Tre**	**PsMBP/Iso**	**PsMBP/Koji**	**PsMBP/Nige**
PDB accession number	9XOR	9XPQ	9XPR	9XPU	9XQ6	9XQ7	9XQL	9XRA	9XRB
Space group	$P2_1$	$P2_1$	$P2_1$	$P2_1$	$P2_1$	$P2_1$	$P2_1$	$P2_1$	$P2_1$
a, *b*, *c*, *β* (Å, °)	69.0, 66.9, 82.8, 96.3	70.5, 64.4, 82.1, 96.7	70.4, 64.8, 82.5, 97.3	70.4, 64.5, 82.4, 96.9	70.9, 64.3, 82.3, 96.4	70.8, 64.2, 81.8, 96.5	69.9, 64.5, 82.3, 96.6	70.1, 65.1, 82.7, 96.6	70.8, 65.1, 82.7, 96.9
Resolution limit (Å)[a]	47.91–1.44 (1.46–1.44)	47.38–1.70 (1.73–1.70)	47.50–1.60 (1.63–1.60)	47.38–1.50 (1.53–1.50)	47.49–1.92 (1.97–1.92)	47.43–1.93 (1.98–1.93)	47.25–1.35 (1.37–1.35)	47.57–2.10 (2.16–2.10)	47.74–2.10 (2.16–2.10)
Measured reflections	609,034	528,946	433,744	775,990	249,306	234,512	724,269	293,353	286,625

Unique reflections	134,536 (6,264)	78,716 (4,056)	96,832 (4,579)	116,395 (5,405)	55,743 (3,672)	52,820 (3,516)	153,321 (7,238)	43,392 (3,469)	43,532 (3,489)
Completeness	99.3 (93.4)	96.2 (95.6)	99.7 (96.3)	99.2 (94.7)	99.0 (97.5)	96.2 (95.8)	96.3 (91.9)	99.8 (97.9)	99.6 (97.9)
Multiplicity	4.5 (4.6)	6.7 (7.0)	4.5 (4.6)	6.7 (6.4)	4.5 (4.6)	4.4 (4.5)	4.7 (4.9)	6.8 (6.9)	6.6 (6.3)
$\langle I/\sigma(I) \rangle$	16.1 (1.6)	25.1 (2.3)	19.7 (1.8)	21.5 (2.5)	12.0 (1.8)	16.7 (1.8)	19.9 (2.1)	24.9 (2.5)	20.0 (1.6)
R_{meas}	0.043 (1.076)	0.033 (0.731)	0.027 (0.794)	0.037 (0.803)	0.053 (0.741)	0.056 (0.968)	0.036 (0.857)	0.047 (0.859)	0.047 (0.963)
R_{pim}	0.027 (0.661)	0.021 (0.455)	0.017 (0.490)	0.023 (0.520)	0.031 (0.435)	0.035 (0.599)	0.022 (0.530)	0.018 (0.322)	0.021 (0.451)
$CC_{1/2}$	0.999 (0.577)	1.000 (0.833)	1.000 (0.779)	1.000 (0.775)	0.999 (0.652)	0.999 (0.717)	0.999 (0.710)	1.000 (0.827)	1.000 (0.686)
Wilson B factor (Å^2)	21.2	27.9	25.3	20.5	35.5	35.4	18.3	46.8	51.8
Refinement									

Final model	396 amino acids (A), 392 amino acids (B), and 901 water molecules	395 amino acids (A), 390 amino acids (B), 509 water molecules, and two maltose molecules	395 amino acids (A), 390 amino acids (B), 649 water molecules, and two maltotriose molecules	398 amino acids (A), 390 amino acids (B), 745 water molecules, and two maltotetraose molecules	397 amino acids (A), 390 amino acids (B), 399 water molecules, and two sucrose molecules	395 amino acids (A), 392 amino acids (B), 408 water molecules, and two trehalose molecules	398 amino acids (A), 390 amino acids (B), 951 water molecules, and two isomaltose molecules	398 amino acids (A), 390 amino acids (B), 242 water molecules, and two kojibiose molecules	398 amino acids (A), 390 amino acids (B), 224 water molecules, and two nigerose molecules
Resolution limit (Å)	35.04–1.44 (1.46–1.44)	37.14–1.70 (1.72–1.70)	37.52–1.60 (1.62–1.60)	28.01–1.50 (1.52–1.50)	40.88–1.92 (1.95–1.92)	33.7–1.93 (1.93–1.90)	35.04–1.35 (1.36–1.35)	39.82–2.10 (2.15–2.10)	42.80–2.10 (2.15–2.10)
Used reflections	134,399 (4,101)	78,522 (2,715)	96,779 (3,062)	115,684 (3,511)	55,703 (2,878)	52,275 (2,832)	153,160 (5,135)	43,345 (2,835)	43,453 (2,812)
Completeness	99.2 (90.3)	97.5 (94.9)	99.6 (94.9)	98.7 (91.4)	98.8 (97.1)	95.0 (93.2)	96.0 (90.7)	99.7 (98.1)	99.5 (97.2)
Average *B*-factor (Å^2)									

Protein	25.1 (A), 27.4 (B)	31.0 (A), 43.6 (B)	28.8 (A), 36.7 (B)	22.5 (A), 29.9 (B)	36.8 (A), 51.2 (B)	34.3 (A), 49.9 (B)	20.9 (A), 25.8 (B)	44.4 (A), 55.7 (B)	49.3 (A), 61.1 (B)
Water	36.7	40.2	38.9	34.0	44.0	42.6	33.6	47.1	50.3
Saccharide		22.9 (A), 31.4 (B)	24.0 (A), 25.7 (B)	17.5 (A), 21.7 (B)	24.5 (A), 37.3 (B)	26.9 (A), 35.7 (B)	16.2 (A), 20.4 (B)	40.0 (A), 46.6 (B)	39.6 (A), 49.9 (B)
R-factor (%)	18.6 (31.1)	20.5 (31.8)	19.2 (28.3)	19.2 (36.8)	19.2 (29.2)	20.0 (36.8)	18.0 (28.0)	19.4 (28.9)	19.7 (28.3)
R_{free} (%)	21.4 (36.1)	23.9 (40.2)	22.9 (32.9)	22.4 (41.1)	24.4 (37.3)	25.5 (42.3)	20.5 (28.5)	25.2 (37.8)	25.6 (32.6)
Root-mean-square deviation									
Bond (Å)	0.005	0.007	0.006	0.006	0.008	0.008	0.005	0.008	0.008
Angle (°)	0.821	0.860	0.826	0.823	0.863	1.032	0.818	0.896	0.925
Ramachandran plot (%)									

Favored region	98.09	97.70	97.57	97.96	97.45	97.19	98.34	96.94	96.56
Allowed region	1.91	2.18	2.43	2.04	2.43	2.68	1.66	2.68	2.93
Outlier region	0	0.13	0	0	0.13	0.13	0	0.38	0.51

[a]Data for the highest resolution shells are given in parentheses.

Table S3. $\boldsymbol{T_{m, app}}$ **values of PsMBP in the presence of various saccharides.**

Saccharide	$T_{m, app}$ (ºC)			Mean $T_{m, app}$ ± S.D. (ºC)	$\Delta T_{m, app}$ (ºC)
Monosaccharide					
None	65.4	65.3	65.0	65.2 ± 0.21	0
Glucose	66.6	66.5	66.6	66.6 ± 0.1	1.4
Mannose	65.5	65.7	65.8	65.7 ± 0.2	0.5
Galactose	65.2	65.3	65.3	65.3 ± 0.1	0.1
Fructose	66.8	66.8	66.9	66.8 ± 0.1	1.6
Xylose	65.6	65.5	65.5	65.5 ± 0.1	0.3
Rhamnose	65.4	65.4	65.4	65.4 ± 0	0.2
GlcNAc	65.1	65.4	65.6	65.4 ± 0.3	0.2
Disaccharide					
None	66.3	66.1	66.0	66.1 ± 0.2	0
Cellobiose	66.2	66.2	66.1	66.2 ± 0.1	0.1
Maltose	77.0	77.1	77.7	77.3 ± 0.4	11.2
Trehalose	75.4	75.2	75.6	75.4 ± 0.2	9.3
Kojibiose	71.1	71.3	71.3	71.2 ± 0.1	5.1
Nigerose	75.1	74.8	75.1	75.0 ± 0.2	8.9
Isomaltose	76.6	76.3	76.7	76.5 ± 0.2	10.4
Sucrose	71.7	71.9	72.4	72.0 ± 0.4	5.9
Melibiose	66.7	66.4	66.2	66.4 ± 0.3	0.3
Lactulose	66.3	66.4	66.6	66.4 ± 0.2	0.3
$(GlcNAc)_2$	66.2	66.0	66.1	66.1 ± 0.1	0
Oligosaccharide					
None	65.1	64.8	64.6	64.8 ± 0.3	0
Maltose	76.4	76.5	76.4	76.4 ± 0.1	11.6
Maltotriose	74.6	74.7	74.6	74.6 ± 0.1	9.8
Maltotetraose	74.3	74.3	74.2	74.3 ± 0.1	9.5
α-Cyclodextrin	69.6	69.7	69.8	69.7 ± 0.1	4.9
γ-Cyclodextrin	66.6	66.4	66.6	66.5 ± 0.1	1.7

Table S4. Interactions between PsMBP and ligands.

PsMBP/Mal							
Glc1		Glc2					
Hydrogen bond		Hydrogen bond					
O1		O1	Ala94, Tyr96				
O2	Glu64	O2	Gly92 Gly93, Ala94				
O3	Lys62, Glu64	O3	Gly92, Gly93				
O4	Lys62, Glu162	O4					
O5		O5					
O6	His216	O6	Glu407				
Hydrophobic contacts	Phe61, Trp291, Trp398, Trp400	Hydrophobic contacts	Trp211				
PsMBP/Tri (chain A)							
Glc1		Glc2		Glc3			
Hydrogen bond		Hydrogen bond		Hydrogen bond			
O1		O1		O1	Glu407		

O2	Glu64	O2		O2	Asp95		
O3	Lys62, Glu64	O3		O3			
O4	Lys62, Glu162	O4		O4			
O5		O5		O5			
O6	His216	O6	Asn215	O6			
Hydrophobic contacts	Phe61, Trp291, Trp398, Trp400	Hydrophobic contacts	Trp211	Hydrophobic contacts	Tyr96 Trp210 Phe401		
PsMBP/Tetra (chain A)							
Glc1		Glc2		Glc3		Glc4	
Hydrogen bond		Hydrogen bond		Hydrogen bond		Hydrogen bond	
O1		O1		O1		O1	Asn115, Trp124
O2	Glu64	O2		O2	Gly97	O2	Trp124
O3	Lys62, Glu64	O3		O3		O3	
O4	Lys62, Glu162	O4		O4		O4	
O5		O5		O5		O5	
O6	His216	O6	Asn215	O6		O6	Asn328

Hydrophobic contacts	Phe61, Trp291, Trp398, Trp400	Hydrophobic contacts	Trp211	Hydrophobic contacts	Tyr96 Trp210 Phe401	Hydrophobic contacts	Tyr96 Phe401
PsMBP/Suc							
Glc1		Fru2					
Hydrogen bond		Hydrogen bond					
O1		O1	Asn215				
O2	Glu64	O2					
O3	Lys62, Glu64	O3	Gly92, Gly93				
O4	Lys62, Glu162	O4	Gly93, Ala94				
O5		O5					
O6	His216	O6	Ala94, Tyr96				
Hydrophobic contacts	Phe61, Trp291, Trp398, Trp400	Hydrophobic contacts	Trp211				
PsMBP/Tre							
Glc1		Glc2					
Hydrogen bond		Hydrogen bond					

O1		O1					
O2	Glu64	O2	Asn215				
O3	Lys62, Glu64	O3	Glu407				
O4	Lys62, Glu162	O4	Asn328				
O5		O5					
O6	His216	O6	Ala94, Tyr96				
Hydrophobic contacts	Phe61, Trp291, Trp398, Trp400	Hydrophobic contacts	Trp211				
PsMBP/Iso (chain A)							
Glc1		Glc2					
Hydrogen bond		Hydrogen bond					
O1		O1	Glu407				
O2	Glu64	O2	Glu407				
O3	Lys62, Glu64	O3					
O4	Lys62, Glu162	O4					
O5		O5					

O6	His216	O6					
Hydrophobic contacts	Phe61,<br>Trp291,<br>Trp398,<br>Trp400	Hydrophobic contacts	Phe61<br>Trp210<br>Trp211				
PsMBP/Koji (chain A)							
Glc1		Glc2					
Hydrogen bond		Hydrogen bond					
O1		O1					
O2	Glu64	O2					
O3	Lys62,<br>Glu64	O3	Asn215				
O4	Lys62,<br>Glu162	O4	Glu407				
O5		O5					
O6	His216	O6	Glu407				
Hydrophobic contacts	Phe61,<br>Trp291,<br>Trp398,<br>Trp400	Hydrophobic contacts	Trp211				
PsMBP/Nige (chain A)							
Glc1		Glc2					
Hydrogen bond		Hydrogen bond					

O1		O1	Glu407				
O2	Glu64	O2	Asn215				
O3	Lys62, Glu64	O3					
O4	Lys62, Glu162	O4					
O5		O5					
O6	His216	O6					
Hydrophobic contacts	Phe61, Trp291, Trp398, Trp400	Hydrophobic contacts	Trp211				

Fig. S1

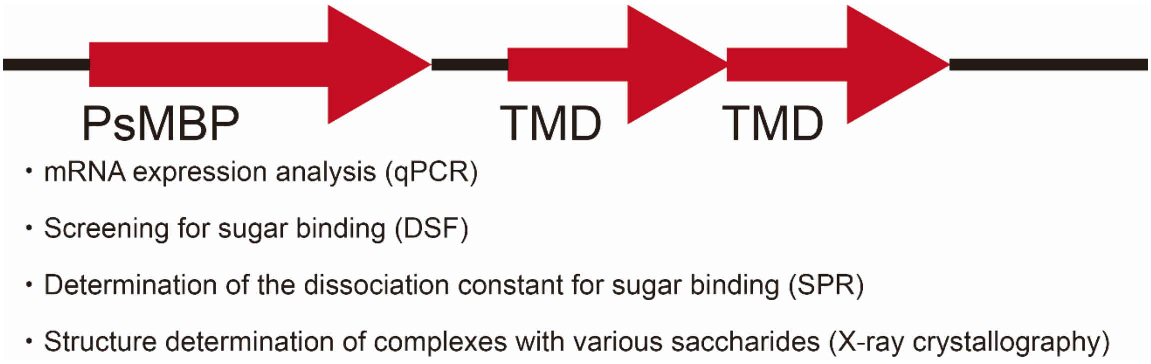

Fig. S1. Genomic context of PsMBP and analytical methods used in this study.

In the draft genome, the SBP gene (PsMBP) is located adjacent to two predicted TMD genes. We confirmed its mRNA expression, identified its sugar-binding specificity, determined the dissociation constants of the corresponding ligands, and elucidated the three-dimensional structures of the resulting complexes.

Fig. S2

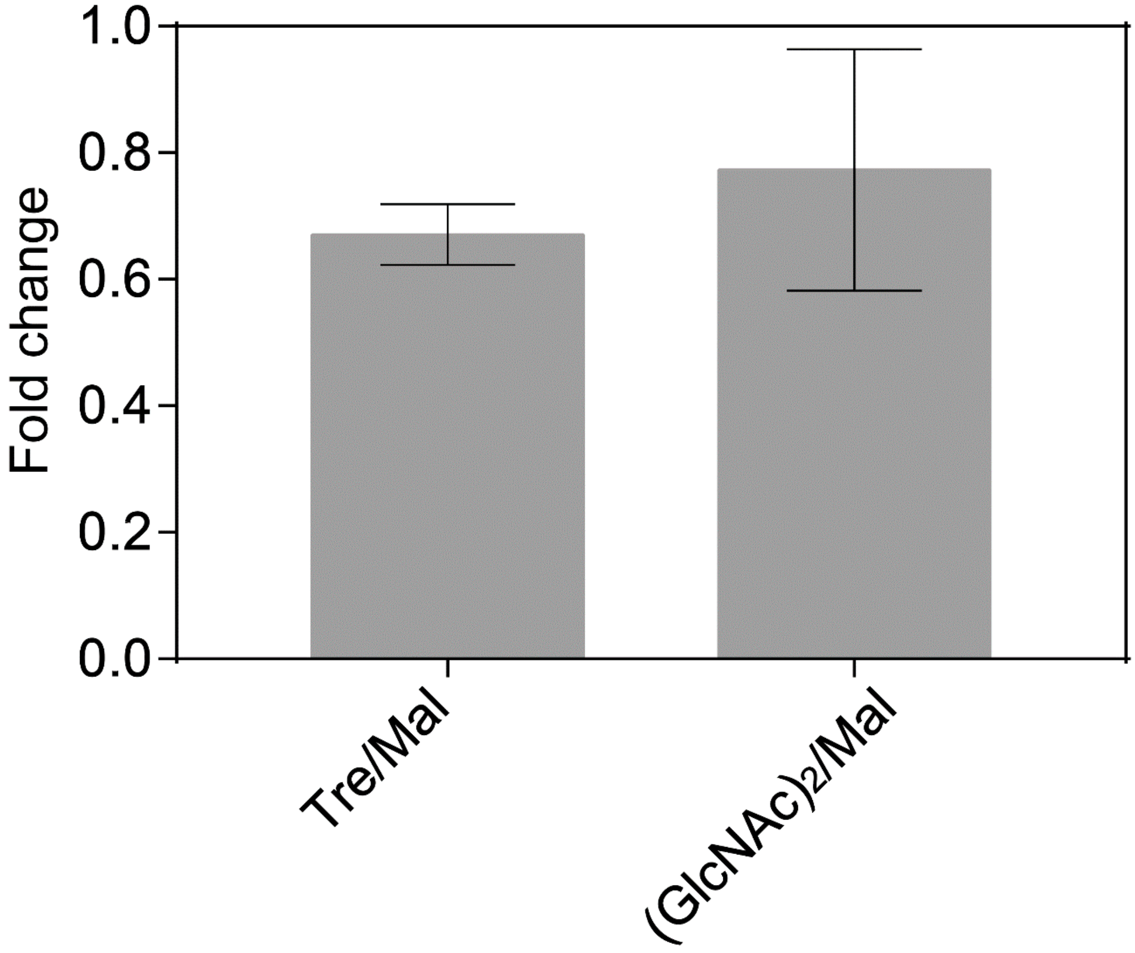

Fig. S2. Fold change in PsMBP gene expression for each saccharide.

The expression levels of the PsMBP gene relative to the 16S rRNA gene in P. FPU-7 cultured in media containing various saccharides (maltose, trehalose, $(GlcNAc)_2$) were evaluated using qPCR. The figure shows the expression levels normalized to 1 using a medium containing maltose as the reference, along with the fold increase in expression levels in media containing trehalose and $(GlcNAc)_2$.

Fig. S3

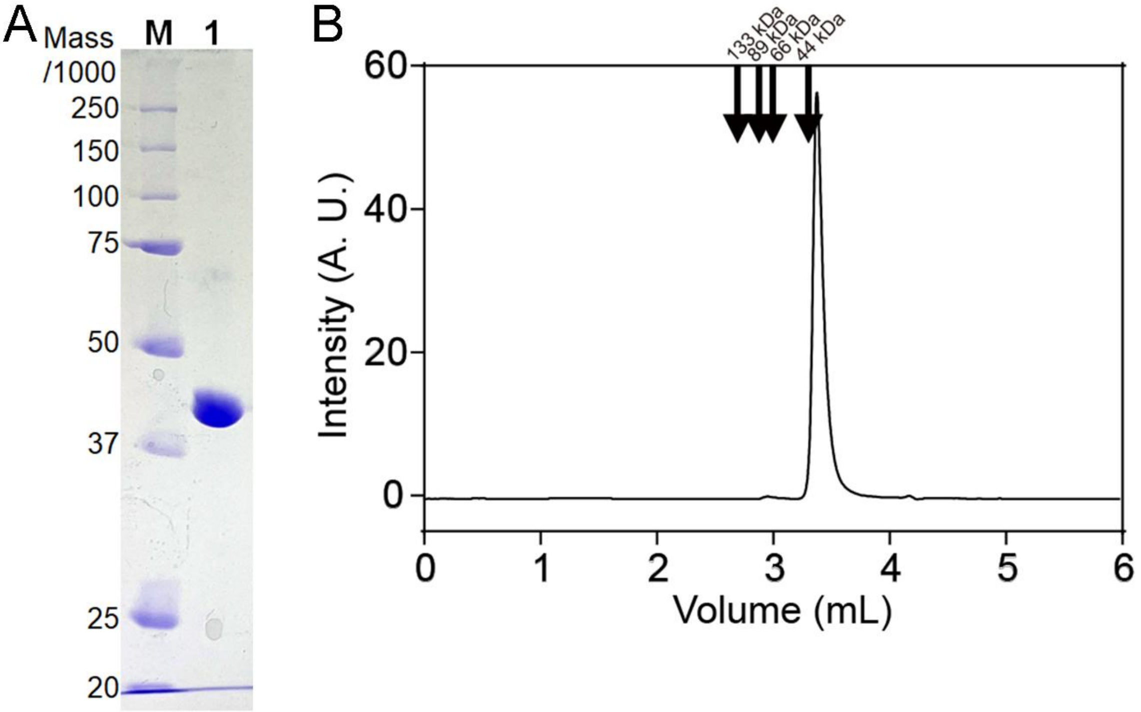

Fig. S3. SDS-PAGE profile and gel filtration chromatogram of PsMBP.

(A) Recombinant PsMBP was expressed in *E*. coli and purified. Proteins were separated by SDS-PAGE and visualized using CBB R-250 staining. Lane M, molecular weight standards (in kDa); lane 1, purified PsMBP (1 μg). The molecular mass of PsMBP was estimated to be 43 kDa, which is lower than the theoretical mass of 47 kDa. (B) The oligomeric state of PsMBP in solution was assessed by gel filtration chromatography. PsMBP eluted as a monomer with an apparent molecular weight of approximately 37 kDa. The elution positions of standard proteins were as follows: bovine serum albumin dimer (133 kDa), bovine serum albumin monomer (66 kDa), ovalbumin dimer (89 kDa), and ovalbumin monomer (44 kDa).

Fig. S4

```
            1                          29                        52
PsMBP       MKREVFFMKWNKIANFSMAAVLLMSVVAGCANKTETPN---NNASGGTGGQSGGKNVTLK   57
NagB1       -------MKGK-RSAVLASVLLLSGLLISCGLDTSFLSSSK-PKSGTAENQEQIKPFTVS   51
NagB2       -------MKKG-LLKTTAAALVLSTALAGCGGTADKNPAGNNGDSAKPAETKSGDSVSLT   52
                   **         :.::*   : .*.  :.         *.     .  . .::.

PsMBP       MFQFKV--EI---AEPLAKLVAEYEKANPGVKIQVESVGGGADYGAALMAKFNSGDKPDI   112
NagB1       LRHTQVRDDVRLRLKMLEDIAQRMEAAVPGLRVELEGVEDKVNRFEKLPAEMAAGNPPKI   111
NagB2       LRHINVRDTAKNTLALLEKVVKKTEAEVPGATFKLDGVEDTVNRDVKLKAEMAAGKPPQI   112
            : : :*          * .:. . *   **  .:::.* . .:    * *:: :*. *.*

PsMBP       FNNGGFSDMEKWQEHLEDLSDQPWVKDLVEV----AKEPMTKDGKLYGQPLNLEGYGFLY   168
NagB1       FDLFGGTDTAKYVKAGRLLELTPILNELGLKDKFPNLQEFTVDGKIYGLPTAYFVEGVFY   171
NagB2       FNLFGGADTQNYAKAGHLLPLNDILKELGLEDKFFELREFTVDGKIYGLPEAGFVEGFYY   172
            *:  * :*  :: :  . *     :::*         . :* ***:** *      *. *

PsMBP       NKDLFAQAGITETPKTLAQLEDAAKKLQAKGVTPFVN--GYGEWWVLGNHFVNIPFAQQK   226
NagB1       NKQIFKQLNV-DVPRRWEDLMDVAAKAKASGFVPFAF--ASSDGWVANMML-NTLWVRT-   226
NagB2       NTKLFADAGITSAPKTWDEFTKALEALKAKNITPIALGGGSGDGWAINMLA-NSLFVAT-   230
            *..:* : .: ..*:   :: ..    :*....*:.   . .: *. .    *  :.

PsMBP       DTNAFIAGLNKGTDKIPGNVQFDNWVKLLDL-QLQYGNKNPLQTDYKTQVTDFATGKAAM   285
NagB1       AGDDSVPGFVRGTRRWTDPDVADGFKRYDTLLKKGYLQEGSLGQKYAEQQYAFREGRAAM   286
NagB2       AGPEAQEGFAKGTTKWTDPAVLDGFKRLKDLKDKGYIDPNVLGLKYSEGQAKFYTGQAAM   290
                   *: :** :  .    *.: :   * .  * : . *  .*      *  *:***

PsMBP       TQQGNWTQLQLTQTN---PNLKVGFLPMPISDDAAANDKLFV-GVPNNWVI--NKNSPNK   339
NagB1       MFDGSWASAALVDAGKTKIAEDIGFFSFPDVGGK--GDGMINGGYSNGYGFSASLNEREK   344
NagB2       LFDGSWATSAILDKDKSTVKDNVGYFRFPNIGGK--GDNLINGGWSNGYGFSSHLNDAEK   348
              :*.*:   : : .      .:*:: :*  ..   .* ::  *  *.: :    *. :*

PsMBP       EEAKKFLNWLATSDTGKKFMVEEFKFIPAFKSVQADEKVLGPLAA---------------   384
NagB1       KAAVEFIKIMYSEEMQKRQ-LKESGILPAMKLSDLSG-VH-PVIR--EMIQASELRQFPA   399
NagB2       KAVKAFIKNFYTLEIQGEA-LGRDNRVPSMKGVPTPA-EAAPLTKAIGEAQASAKAAFPA   406
            : .  *:: : : :   .  .   :*::*          *:

PsMBP       -DIIKYSKDNKTLSWNWFKFPGGEATSKKFAATIQAYVAKQKTKDQMLTEFQQTWDSLKK   443
NagB1       FDSIVQAKVRETLEMCMQELIGGR-----------------MTVEQVLDKMQKVQEDANR   442
NagB2       FDALVQPKVKVTLEQSVQELLGGQ-----------------LTPEKLVEKMQKVQDEANA   449
             * :   * . **.    :: **.                  * :::: ::*:. :. :

PsMBP       ----      443
NagB1       DMKK      446
NagB2       GK--      451
```

Fig. S4. Amino acid sequence alignment of PsMBP, NagB1, and NagB2.

The amino acid sequence of PsMBP was aligned with those of NagB1 and NagB2 using ClustalW.

A signal peptide was predicted at the N-terminus of PsMBP (residues Met1–Gly29), and a lipid

modification site was identified at Cys30. The sequence segments of NagB1 and NagB2 highlighted in blue, excluding the predicted signal peptide, have not been resolved by X-ray crystallography (NagB1: Cys22–Phe48; NagB2: Cys22–Ser45). To obtain crystals, we prepared a truncated PsMBP lacking the corresponding region (Cys30–Lys52, highlighted in red) and used it for crystallization.

Fig. S5

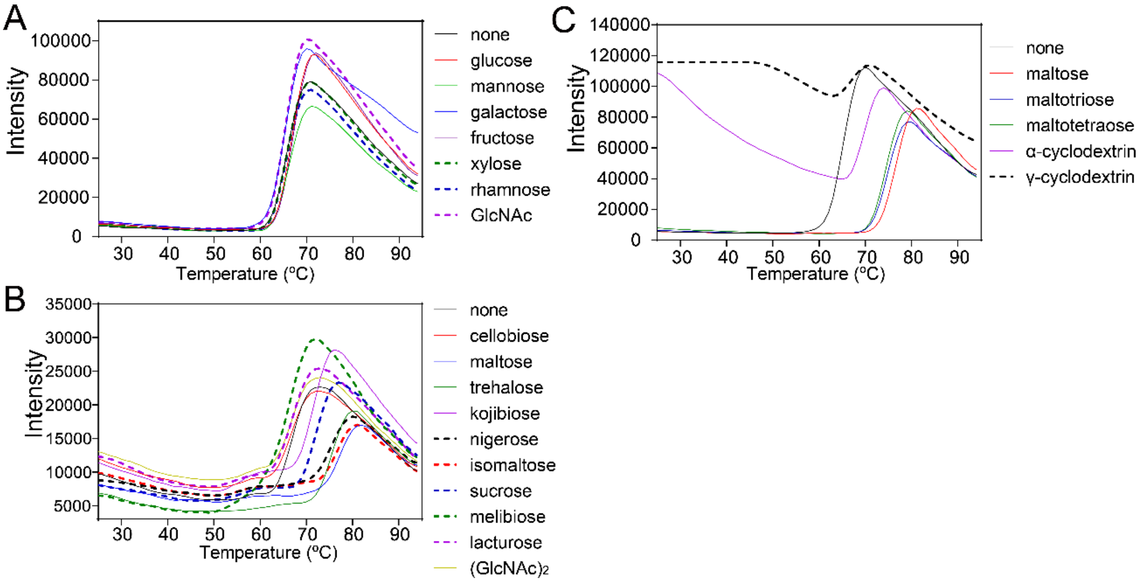

Fig. S5. Differential scanning fluorimetry analysis of PsMBP in the presence of various saccharides.

(A) Representative melting curves of PsMBP from three independent experiments in the presence of the following monosaccharides: none (black solid line), glucose (red solid line), mannose (green solid line), galactose (blue solid line), fructose (purple solid line), xylose (green dashed line), rhamnose (blue dashed line), and GlcNAc (purple dashed line). (B) Representative melting curves of PsMBP from three independent experiments in the presence of the following disaccharides: none (black solid line), cellobiose (red solid line), maltose (blue solid line), trehalose (green solid line), kojibiose (purple solid line), nigerose (black dashed line), isomaltose (red dashed line), sucrose (blue dashed line), melibiose (green dashed line), lactulose (purple dashed line), and $(GlcNAc)_2$ (yellow dashed line). (C) Representative melting curves of PsMBP

from three independent experiments in the presence of the following oligosaccharides: none (black solid line), maltose (red solid line), maltotriose (blue solid line), maltotetraose (green solid line), α-cyclodextrin (purple solid line), and γ-cyclodextrin (black dashed line). Background fluorescence increased in the presence of α-cyclodextrin and γ-cyclodextrin.

Fig. S6

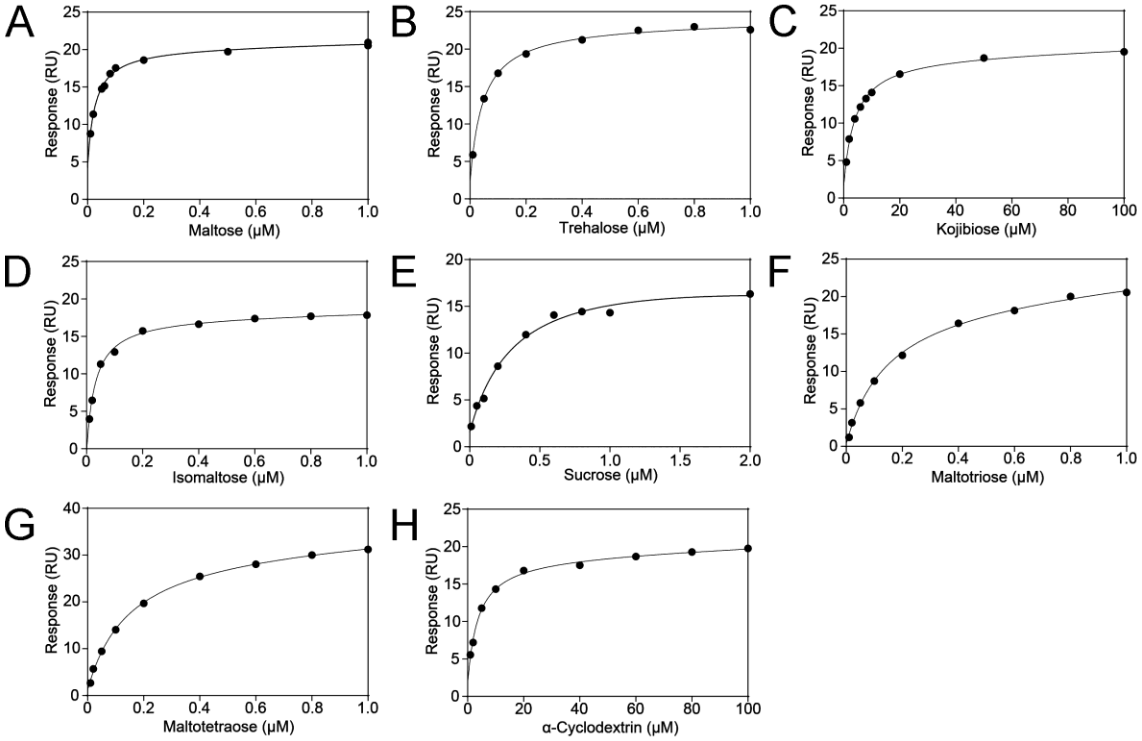

Fig. S6. Binding affinity of PsMBP for various saccharides.

SPR binding assays were conducted using a range of concentrations for the following saccharides: maltose (A), trehalose (B), kojibiose (C), isomaltose (D), sucrose (E), maltotriose (F), maltotetraose (G), and α-cyclodextrin (H). Response units, reflecting the extent of complex formation at each concentration, were plotted. Equilibrium dissociation constants (K_D) were calculated by fitting the data to a one-site binding model (R = [S] R_{max}/ (K_D + [S])), where R represents the response units and [S] the saccharide concentration.

Fig. S7

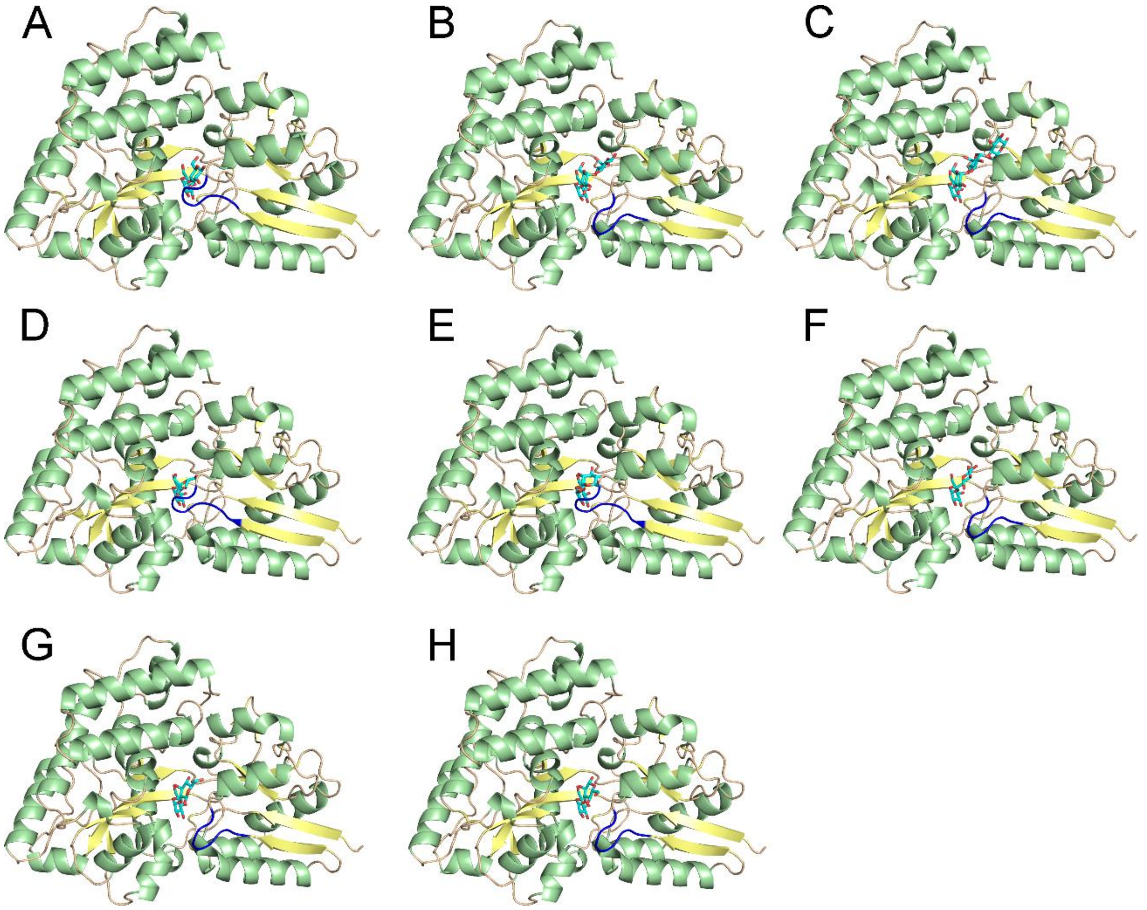

Fig. S7. Overall structures of PsMBP bound to various saccharides.

Ribbon representations of PsMBP in complex with different saccharides: maltose (PsMBP/Mal) (A), maltotriose (PsMBP/Tri) (B), maltotetraose (PsMBP/Tetra) (C), sucrose (PsMBP/Suc) (D), trehalose (PsMBP/Tre) (E), isomaltose (PsMBP/Iso) (F), kojibiose (PsMBP/Koji) (G), and nigerose (PsMBP/Nige) (H).

Fig. S8

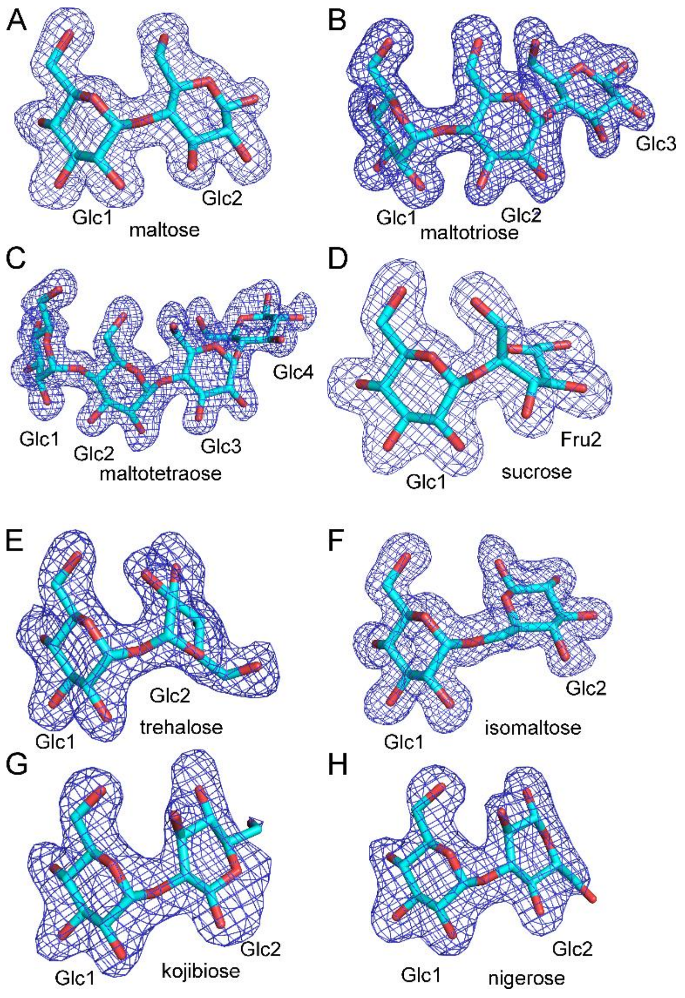

Fig. S8. Electron density maps around the ligands.

Electron densities (blue) surrounding the ligands (cyan stick model) are shown as composite omit map (2mF_o-DF_c), calculated with simulated annealing and contoured at the 1.0 σ level: maltose (PsMBP/Mal) (A), maltotriose (PsMBP/Tri) (B), maltotetraose (PsMBP/Tetra) (C), sucrose (PsMBP/Suc) (D), trehalose (PsMBP/Tre) (E), isomaltose (PsMBP/Iso) (F), kojibiose (PsMBP/Koji) (G), and nigerose (PsMBP/Nige) (H).

Fig. S9

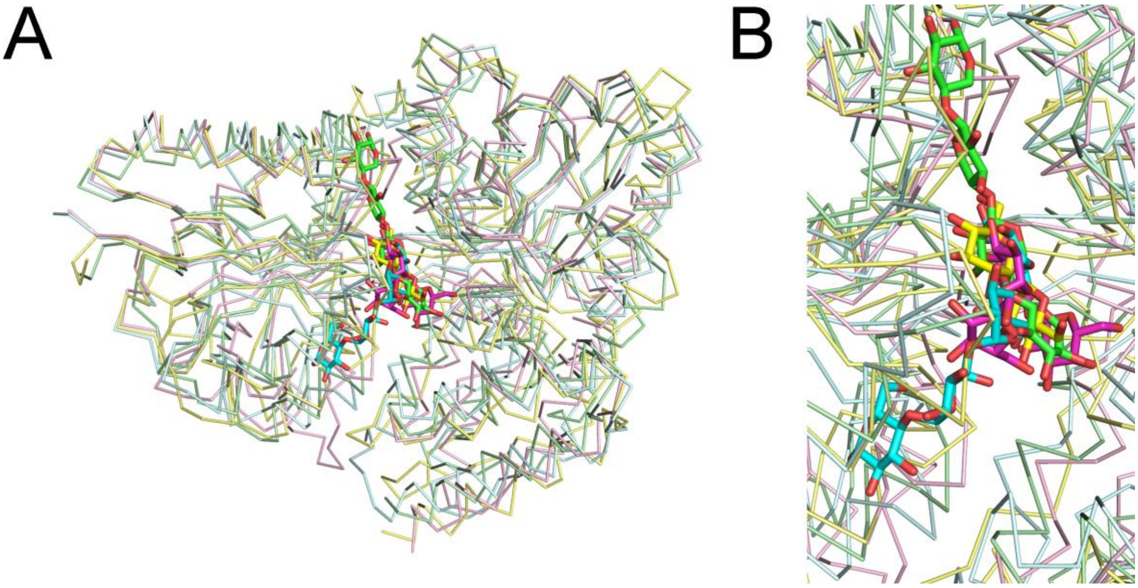

Fig. S9. Structural comparisons of PsMBP with other SBPs.

(A) Superimposed ribbon diagrams of PsMBP bound to maltotetraose (PsMBP/Tetra; cyan) and three representative oligosaccharide-binding SBPs: the xylo-oligosaccharide-binding protein (PDB ID: 3ZKK; green), the raffinose/panose-binding protein (PDB ID: 4ZS9; magenta), and the trehalose/maltose-binding protein (TlMBP) (PDB ID: 1EU8; yellow). Bound oligosaccharide ligands are shown as stick representations. (B) Close-up views of the ligand-binding sites corresponding to the structures shown in panel A.

Fig. S10

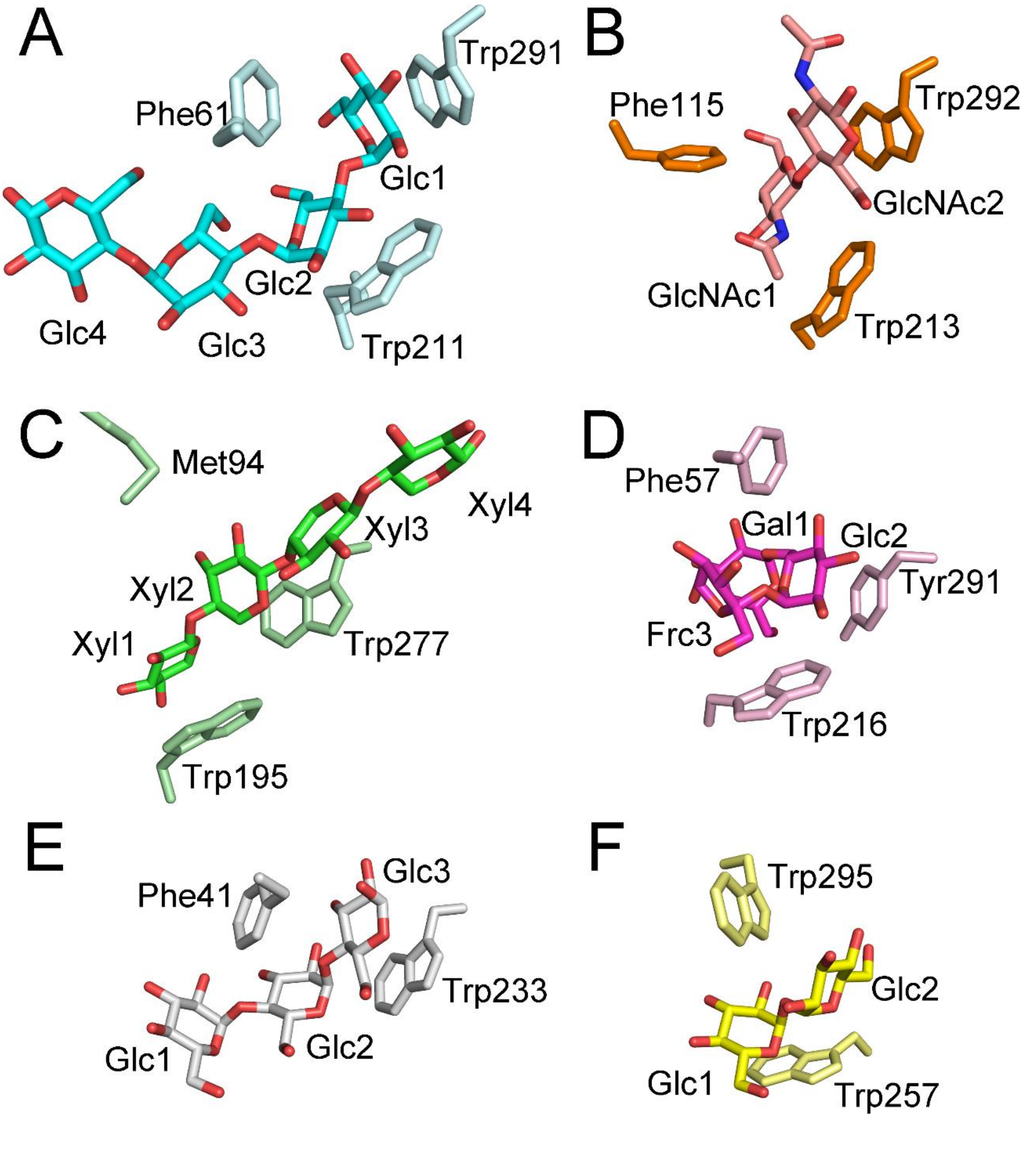

Fig. S10. Conservation of a phenylalanine and two tryptophan residues in the PsMBP binding site.

(A) The α-(1→4) glycosidic bond between Glc1 and Glc2 in PsMBP interacts with Phe61. Glc1 and Glc2 interact with Trp211 and Trp291 through CH/π interactions. (B) The β-(1→4) glycosidic

bond between GlcNAc1 and GlcNAc2 in NagB1 (PDB ID: 7EHU) interacts with Phe115. GlcNAc1 and GlcNAc2 interact with Trp213 and Trp292 through CH/π interactions. (C) In the xylo-oligosaccharide-binding protein from *Bifidobacterium animalis* (PDB ID: 3ZKK), the phenylalanine residue is absent, although two tryptophan residues (Trp195 and Trp277) are present. (D) In the raffinose/panose-binding protein from *Bifidobacterium animalis* (PDB ID: 4ZS9), three residues—Phe57, Trp216, and Trp291—are present. (E) In the maltose-binding protein from *Thermotoga maritima* (TmMBP) (PDB ID: 2FNC), two residues—Phe41 and Trp233—are present. (F) In the trehalose/maltose-binding protein from *Thermococcus litoralis* (TlMBP) (PDB ID: 1EU8), a tryptophan residue (Trp295) is present in place of the phenylalanine residue.
